# Adaptation to HLA-associated immune pressure over the course of HIV infection and in circulating HIV-1 strains

**DOI:** 10.1371/journal.ppat.1010965

**Published:** 2022-12-16

**Authors:** Eric Alves, Marwah Al-Kaabi, Niamh M. Keane, Shay Leary, Coral-Ann M. Almeida, Pooja Deshpande, Jennifer Currenti, Abha Chopra, Rita Smith, Alison Castley, Simon Mallal, Spyros A. Kalams, Silvana Gaudieri, Mina John

**Affiliations:** 1 School of Human Sciences, The University of Western Australia, Perth, Western Australia, Australia; 2 Institute for Immunology and Infectious Diseases, Murdoch University, Perth, Western Australia, Australia; 3 Division of Infectious Diseases, Department of Medicine, Vanderbilt University Medical Center, Nashville, Tennessee, United States of America; 4 Department of Clinical Immunology, Royal Perth Hospital, Perth, Western Australia, Australia; Johns Hopkins University School of Medicine, UNITED STATES

## Abstract

Adaptation to human leukocyte antigen (HLA)-associated immune pressure represents a major driver of human immunodeficiency virus (HIV) evolution at both the individual and population level. To date, there has been limited exploration of the impact of the initial cellular immune response in driving viral adaptation, the dynamics of these changes during infection and their effect on circulating transmitting viruses at the population level. Capturing detailed virological and immunological data from acute and early HIV infection is challenging as this commonly precedes the diagnosis of HIV infection, potentially by many years. In addition, rapid initiation of antiretroviral treatment following a diagnosis is the standard of care, and central to global efforts towards HIV elimination. Yet, acute untreated infection is the critical period in which the diversity of proviral reservoirs is first established within individuals, and associated with greater risk of onward transmissions in a population. Characterizing the viral adaptations evident in the earliest phases of infection, coinciding with the initial cellular immune responses is therefore relevant to understanding which changes are of greatest impact to HIV evolution at the population level. In this study, we utilized three separate cohorts to examine the initial CD8^+^ T cell immune response to HIV (cross-sectional acute infection cohort), track HIV evolution in response to CD8^+^ T cell-mediated immunity over time (longitudinal chronic infection cohort) and translate the impact of HLA-driven HIV evolution to the population level (cross-sectional HIV sequence data spanning 30 years). Using next generation viral sequencing and enzyme-linked immunospot interferon-gamma recall responses to peptides representing HLA class I-specific HIV T cell targets, we observed that CD8^+^ T cell responses can select viral adaptations prior to full antibody seroconversion. Using the longitudinal cohort, we uncover that viral adaptations have the propensity to be retained over time in a non-selective immune environment, which reflects the increasing proportion of pre-adapted HIV strains within the Western Australian population over an approximate 30-year period.

## Author summary

Human immunodeficiency virus (HIV) utilizes numerous mechanisms to evade the host’s immune response, including the selection of specific polymorphisms, termed viral adaptations, which can alter recognition and elimination by cytotoxic T-lymphocytes (CTLs). This study utilized three unique treatment-naïve cohorts to characterize the initial CTL response to HIV, track the dynamics of viral adaptation during early infection, and examine how viral adaptations shape the circulating stains within one population over decades of time. Using an acute HIV-infected cohort, we applied genetic and cellular assays to identify evidence of anti-HIV CTL immune responses prior to antibody seroconversion providing an early selection environment. In the longitudinally-sampled chronic cohort, we identify viral adaptations already evident by the first year of infection that do not revert, and are stably retained over time. Finally, we present evidence for viral adaptations accumulating in the circulating strains of the Western Australian population over a thirty year period.

## Introduction

Human immunodeficiency virus type 1 (HIV-1) has an enormous capacity to continuously diversify via its rapid and error-prone replication. Newly generated mutations can accumulate within the viral quasispecies due to genetic drift and/or a fitness advantage in the host immune environment. The latter mutations achieve greater viral fitness by allowing resistance against host immune responses or antiretroviral therapy (ART). Anti-viral cytotoxic T-lymphocyte (CTL) responses, restricted by human leukocyte antigen (HLA) class I alleles, constitute one of the major host selective forces on HIV evolution, particularly during the first 12 months of infection [1, 2]. Numerous studies have reported HIV mutational escape from CTL-mediated pressure via the partial or complete disruption of T cell receptor (TCR)-peptide-HLA interactions [3–6]. These adaptations become apparent as mutational “footprints” in the form of site-specific associations between viral polymorphisms and HLA alleles, similar to what is seen with ART resistance mutations and specific drugs [7–9]. Importantly, viral adaptations have been confirmed over the past two decades to be reproducible based on the HLA alleles of the host, and mark viral sites under strong *in vivo* immune pressure [9–11]. Once selected, viral adaptations, which have been estimated to account for variation at 24%-56% of sites within the Gag, Pol and Nef HIV proteins, can be transmitted between individuals. A high level of viral adaptation to the host’s HLA repertoire is associated with impaired viral immunogenicity, elevated viral loads and accelerated CD4^+^ T cell decline [12].

Although transmitted viral adaptations are often maintained in HLA-matched recipients [13], the situation of HLA-mismatched recipients is complex and less clear. On one hand, various viral adaptations are reported to undergo reversion to the wildtype state upon transmission to a new HLA-mismatched host, likely to restore efficient viral replication [8, 14–16]. On the other hand, studies have shown evidence of HIV adaptations within the circulating strains of a host population [7, 17–21], demonstrating that HIV strains harboring HLA-specific adaptations have the capacity to increase in frequency within an HLA-diverse population. In the context of vaccine design, population-level HIV adaptation in response to HLA-associated immune pressure can diminish vaccine efficacy within and between HLA-diverse populations [22].

Elimination of the latent reservoir–a stable pool of long-lived cells harboring integrated proviral HIV DNA [23, 24]–is a key objective of HIV cure strategies. Given that seeding of HIV into these reservoirs begins rapidly following infection [25] and persists throughout active HIV replication [26], the viral quasispecies contained within these latent compartments has been shown to be genetically diverse [27–29] and contain host-associated viral adaptations [30]. As such, reactivation of the latent reservoir for curative purposes may result in the emergence of adapted HIV variants, which in turn, could undermine treatment efficacy. Therefore, a detailed understanding of viral adaptation dynamics over the entire course of HIV infection is key to elucidate the importance of HLA-associated viral adaptations within subjects and in circulating strains of the virus.

Here, we sought to evaluate the initial HIV-specific CTL response in an acute, ART-naïve cohort using enzyme-linked immunospot (ELISpot) interferon-gamma (IFNγ) recall responses to subject-HLA-specific HIV peptides, to confirm previous work by others identifying preferential targeting of T cell epitopes in Gag, Pol and Nef [31–34]. We then use this information to assess, using deep sequencing methods, how HLA-associated T cell-mediated immune pressure may drive the emergence of viral adaptations in Gag, Pol and Nef over the course of HIV infection in a historical ART-naïve longitudinal cohort. Furthermore, confirmation of the long-term dynamics of these adaptations at a population-level in Pol was obtained by examining circulating HIV sequences over a time span of 30 years in the Western Australian population.

## Results

### HIV-specific CTL responses in subjects pre-seroconversion are likely to exert early selection pressure on the virus

**Fig 1:**
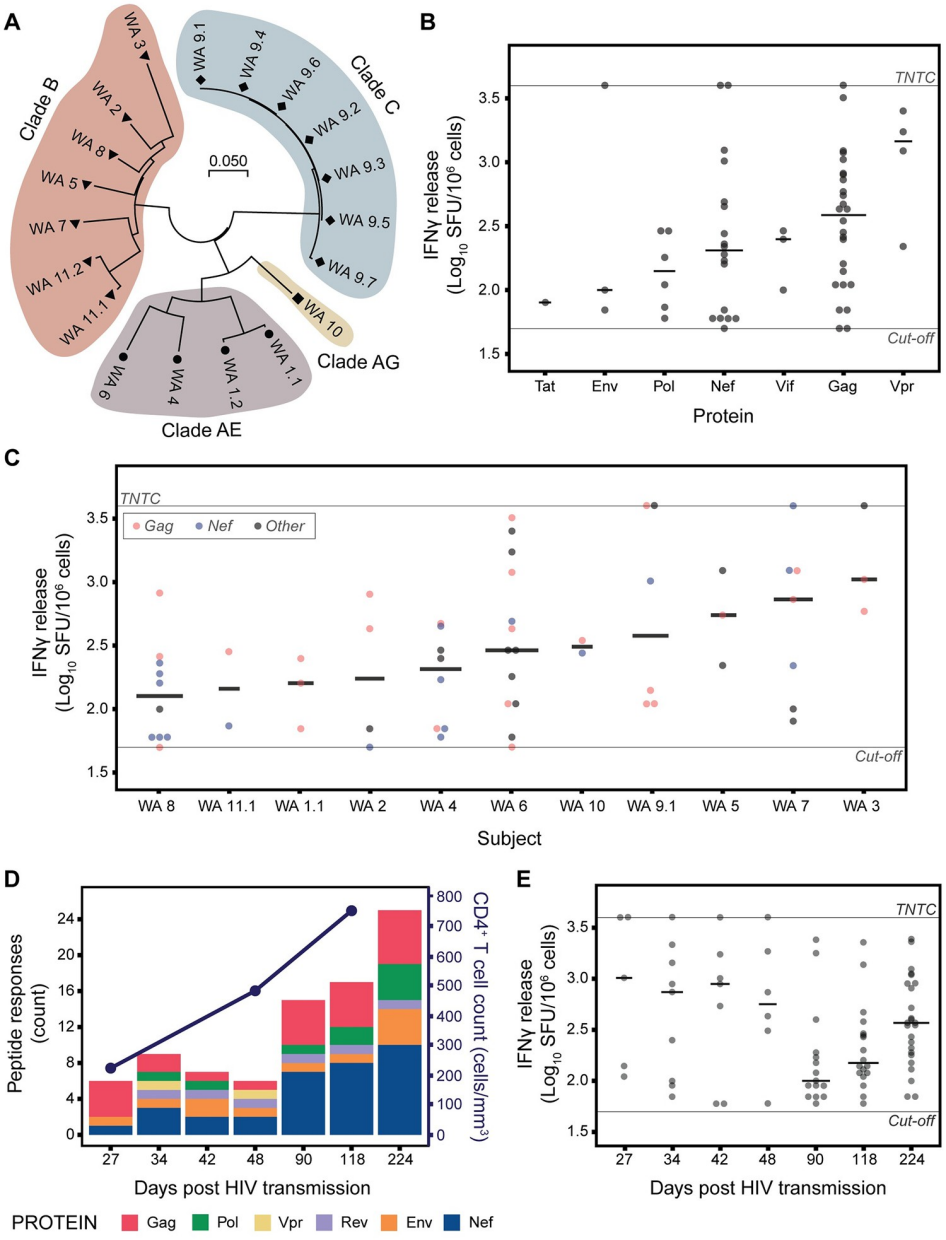
IFNγ responses to HLA-associated HIV-specific CTL epitopes are detected in HIV-1 infected subjects prior to full seroconversion. **A)** Evolutionary analysis of combined Gag, Pol and Nef consensus sequences for each subject supports infection with clade B for six of the 11 subjects and within subject clustering for longitudinal samples. Phylogenetic tree constructed using the Maximum Likelihood method with the Tamura-Nei model. **B & C)** IFNγ ELISpot responses for peptides tested on subject PBMCs collected at the earliest sampling date. Protein **(B)** and subject **(C)** responses are in ascending order according to median IFNγ release (black lines). **D)** IFNγ ELISpot responses increase over the course of acute infection in subject WA9 and correspond with improved CD4^+^ T cell counts. **E)** Evidence of high magnitude, narrow IFNγ ELISpot responses during the first month post presentation. T cells tested three months post transmission or later exhibit lower magnitude and broad IFNγ responses. TNTC (too numerous to count) denotes upper limit of assay (3.6 log_10_ SFU/10^6^ cells). Cut-off denotes lower threshold for positive responses (1.7 log_10_ SFU/10^6^ cells).

To examine CTL-mediated immunity during acute/early HIV infection, peripheral blood mononuclear cell (PBMC) samples collected from 11 acute/early-stage HIV infected subjects (*n* = six clade B, three clade AE, one clade C and one clade AG; Fig 1A) with known/inferred HIV transmission dates (acute WA cohort; Table 1) were assessed for IFNγ responses following stimulation with individualized peptide sets. These peptide sets comprised peptides representing specific HIV clade B-based CTL epitopes–previously shown to be clade cross-reactive–associated with one or more of the HLA alleles carried by each subject (S1 Table). The viral targets of the early host response were determined by analyzing the first samples collected from each subject (median 46, range 27–177 days post HIV transmission; median Fiebig stage IV, range II-VI). HIV-specific CTL responses in subjects prior to antibody seroconversion were detected as early as Fiebig stage II in subjects WA9 and WA10.

**Table 1:** Clinical and immunogenetic characteristics of the acute WA cohort.

*Subject ID*	*Sex*	*Age at initial sample (y)*	*Clade*	*Fiebig stage*	*Days post transmission*^ *of Fiebig stage test*	*HLA-A*		*HLA-B*		*HLA-C*		*Sample ID*	*Days post transmission of sample collection* ^	*CD4*^+^ *T cell count (cells/mm*^*3*^*)*	*Viral load (log*_*10*_ *copies/mL)*^!^
WA1	M	39	AE	IV	43	02	03	44	55	02	03	WA1.1	46	455	5.34
												WA1.2	65		
WA2	M	47	B	IV	31	02	03	07	40	03	07	WA2	48	408	
WA3	M	39	B	IV	31	01	02	08	44	05	07	WA3	43	260	6
WA4	M	26	AE	IV	31	02	11	15	38	07	08	WA4	46	320	
WA5^#^	M	45	B	III	120	02	24	39	55	01	07	WA5	177	738	6
WA6	M	18	AE	IV	64	02	11	07	13	03	07	WA6	84	484	4.61
WA7	F	29	B	IV	31	01	29	08	44	07	16	WA7	47	280	4.77
WA8	M	23	B	VI	101	02	24	15	40	04	15	WA8	117	368	
WA9*	F	43	C	II	23	01	02	07	08	07	07	WA9.1	27	225	6
												WA9.2	34		
												WA9.3	42		
												WA9.4	48	486	5.01
												WA9.5	90		
												WA9.6	118	756	4.47
												WA9.7	224		
WA10	M	37	AG	II	30	01	03	08	18	01	07	WA10	37	330	
WA11	M	24	B	IV	31	02	24	38	56	01	07	WA11.1	31	117	6
												WA11.2	38		

^Inferred (based on Fiebig staging) or known date of HIV transmission;

^!^Upper limit of viral load assay is 6 log_10_(copies/mL);

^#^PEP administered 2 days post transmission resulted in delayed seroconversion;

*WA9 is heterozygous for CCR5-Δ32.

Most responses were directed against peptides representing epitopes within the HIV proteins Gag (26/61 peptides) and Nef (18/61 peptides) (Fig 1B). Furthermore, IFNγ responses to Gag peptides were detected in all (11/11) subjects, and to Nef peptides in 72% (8/11) of subjects (Fig 1C), supporting previous literature [34] that these proteins are immunogenic during acute and early HIV infection. In general, CTL responses recorded from the first sample of each subject targeted multiple proteins with a median of four (range 2–13) responses per subject (median magnitude 250 SFU/10^6^ cells), including four responses at the upper limit of detection for the assay of 4000 SFU/10^6^ cells, of which two were from WA9 at Fiebig stage II (Fig 1C).

### Longitudinal case study of acute-stage infection reveals a transition in CTL targeting over time and early emergence of viral adaptations

From the acute WA cohort, subject WA9 had plasma and PBMC samples available for longitudinal examination of CTL responses and viral evolution during acute-stage infection. This subject presented with clade C HIV infection 23 days after known heterosexual transmission, with a positive p24 antigen and negative antibodies by western blot, indicating acute HIV infection at Fiebig stage II (pre-seroconversion). Testing three months later (118 days post transmission) showed a fully positive western blot with a positive p31 band, indicative of Fiebig stage VI (full seroconversion). PBMC and plasma samples were collected weekly for the first month from presentation (27, 34, 42 and 48 days post transmission) and then on days 90, 118 and 224.

PBMC samples were assessed for IFNγ responses following stimulation with peptides representing known HLA class I-associated CTL epitopes matching the HLA alleles present in the subject. Of the 64 peptides evaluated, CTL responses were detected to 29 (45%), with a median of nine (range 6–25) peptide responses over the seven time points collected (Fig 1D). Four of the six responses detected in the first PBMC sample were directed against Gag peptides. Responses increased post seroconversion, peaking at 25 responses across multiple proteins by day 224 prior to therapy initiation on day 231 (Fig 1E). This broadening CTL response over time was observed during an HIV viral load decline from >1 million HIV RNA copies/mL at initial sampling, to 29,512 HIV RNA copies/mL at full seroconversion. When responses in the first two months (28 peptides, day 27–48 post transmission; denoted as a conservative pre-seroconversion period and at early Fiebig staging) were compared with later responses (57 peptides, three months post transmission or later; encompassing the period of full seroconversion), Gag responses were similar for both time periods (28% [8/28] during the first month post presentation, and 28% [16/57] at three months post transmission or later), whilst Nef responses increased from 28% (8/28) to 43% (25/57) in the same interval. Median magnitude of responses for the first month post presentation and at early Fiebig staging (median 740, range 60–4000 SFU/10^6^ cells) was seen to be higher compared to at full seroconversion (median 200, range 60–2440 SFU/10^6^ cells), exhibiting a transitional change from high magnitude, narrow responses to lower magnitude, broad responses (Fig 1E).

The early immune responses detected in this case at Fiebig stage II combined with decreasing HIV viral load up to Fiebig stage VI suggests the presence of immune pressure on the virus that could lead to viral adaptation. Paired longitudinal analysis of ELISpot and deep sequencing data identified a single site at position 357 in Gag, which transitions from serine (S) to glycine (G) by Fiebig stage VI. This site lies within the immunodominant HLA-B*07-associated GL9 (GP_HKARVL, S3G) epitope and is a known adaptation site previously described to result in a high-avidity CTL response, driving cells to exhaustion [17, 35, 36]. Subject WA9, who carries HLA-B*07, also responded to the adapted form of the epitope (S1 Fig), in keeping with previous studies demonstrating maintained or increased recognition of the adapted immunodominant GL9 epitope as an alternative viral adaptation mechanism [36, 37].

### High rate of nucleotide change over time highlights the mutational flexibility of HIV proteins

**Fig 2:**
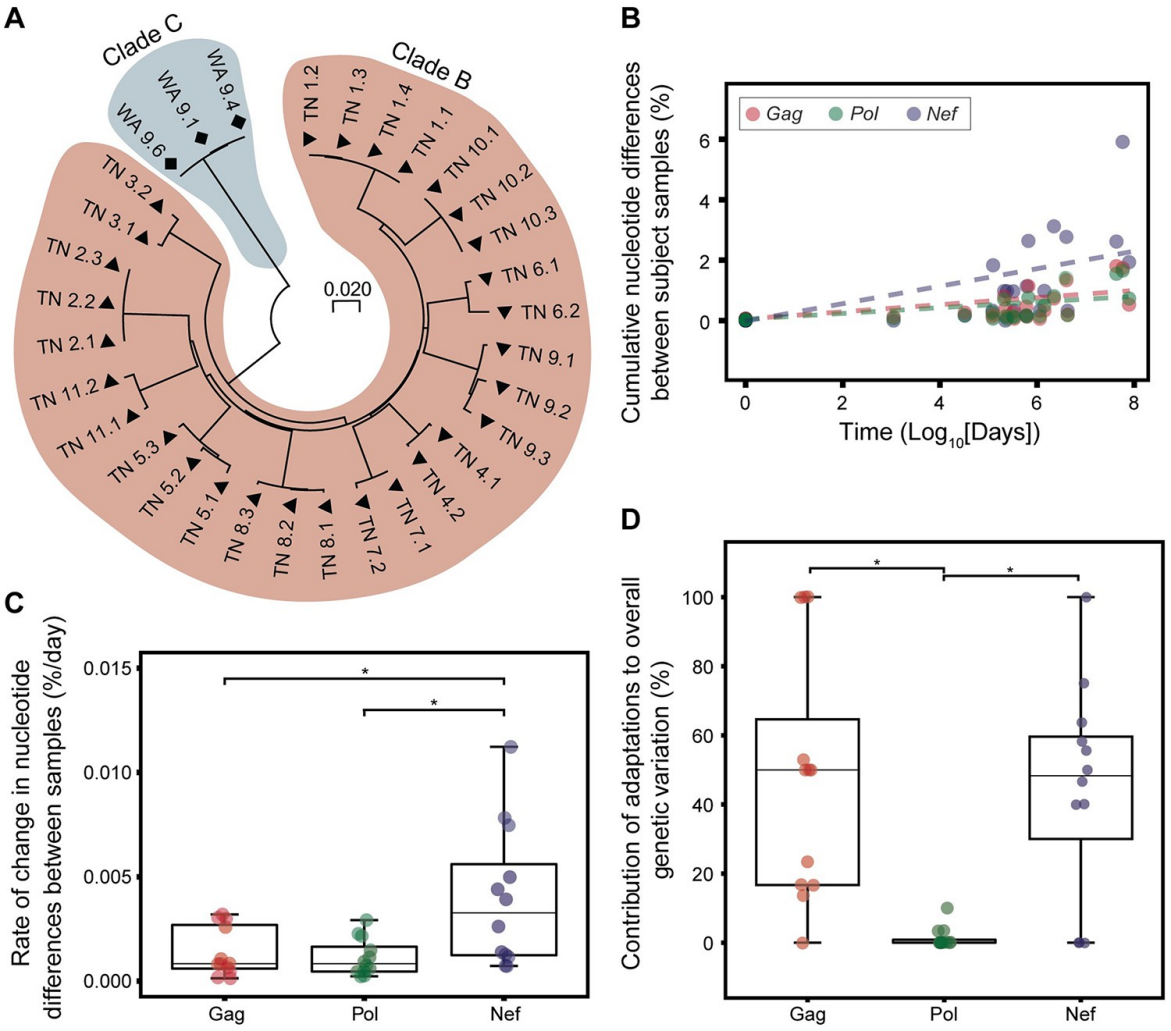
The HIV proteome, particularly Nef, exhibits significant nucleotide variability over time. **A)** Evolutionary analysis of Gag, Pol and Nef majority sequences illustrates clade and longitudinal sample clustering. Phylogenetic tree constructed using the Maximum Likelihood method with the Hasegawa-Kishino-Yano model. **B)** Nucleotide variation in Gag, Pol and Nef all exhibit a significant positive correlation with time (*p* < 0.001 for all three proteins; mixed effects linear regression model). **C)** Nef demonstrates the greatest variation at the nucleotide level compared to Gag (*, *p* = 0.032) and Pol (*, *p* = 0.021), as measured using rate of change per day. **D)** Viral adaptations contribute significantly to the genetic variation in Gag (*, *p* = 0.022) and Nef (*, *p* = 0.036), compared to Pol. Analyses in (C) and (D) using Friedman test and Holm-corrected Wilcoxon test.

Next, given the preferential targeting of T cell epitopes in Gag, Pol and Nef in the WA cohort, we sought to assess the overall rate of nucleotide variation in these proteins over time. Longitudinal deep sequencing of HIV quasispecies was obtained from subject WA9 and 11 additional ART-naïve HIV-infected subjects (TN cohort; Table 2). Longitudinal sample collection for the TN cohort started at a median 245 (range 152–360) days from the last negative HIV test or known year of infection (conservative estimate for calculation set as January 1^st^) and spanning a median of 468 (range 163–2,676) days. The proteins Gag, Pol and Nef constitute three out of four classical targets for vaccine development [38–40]. The fourth protein Env was excluded from this analysis as it is the dominant target for antibodies, and CTL-associated viral adaptations may be confounded with antibody binding-associated mutations [41]. Phylogenetic analysis of the consensus sequences obtained for all three proteins supported longitudinal sampling and clade-related clustering (Fig 2A). As expected, genetic variation in Gag, Pol and Nef nucleotide sequences for each subject were positively correlated with time (calculated as time since previous sample collection; *p* < 0.001 for all three proteins; mixed-effects linear regression model; Fig 2B). Moreover, when normalizing for time between initial and final sample collections using rate of change, Nef exhibited a significantly greater degree of nucleotide variation over time in this cohort (*X*^*2*^[2] = 8.167, *p* = 0.017; Friedman test). This corresponded to a 2.8-fold and 3.5-fold greater mean rate of change in Nef compared to Gag (*p* = 0.032; Holm-corrected Wilcoxon test) and Pol (*p* = 0.021), respectively (Fig 2C). No significant difference was identified between the rate of overall genetic variation in Gag and Pol (*p* = 0.339), although Gag had a minor 1.2-fold greater mean rate of change when compared to Pol. The same pattern of overall nucleotide change was also observed when examining synonymous and nonsynonymous variation individually (S2 Fig).

**Table 2:** Clinical and immunogenetic characteristics of TN cohort.

*Subject ID*	*Ethnicity*	*Age at initial sample (y)*	*HLA-A*		*HLA-B*		*HLA-C*		*Sample ID*	*Days since initial sample*	*CD4*^+^ *T cell count (cells/mm*^*3*^*)*	*Viral load (log*_*10*_ *copies/mL)*
TN1	W	38	03:01	32:01	18:01	27:05	02:02	12:02	TN1.1		666	3.52
									TN1.2	251	832	3.65
									TN1.3	426	986	4.15
									TN1.4	756	720	3.57
TN2	W	36	02:01	02:01	15:01	51:01	03:03	14:02	TN2.1		740	2.21
									TN2.2	210	690	4.09
									TN2.3	322	903	3.79
TN3	W	40	11:01	32:01	27:05	51:01	02:02	14:02	TN3.1		731	2.80
									TN3.2	2,676	1,124	3.57
TN4	W	37	01:01	03:02	08:01	08:01	07:01	07:02	TN4.1		512	3.42
									TN4.2	247	693	4.26
TN5	AA	20	02:02	68:02	15:03	45:01	02:02	16:01	TN5.1		432	4.43
									TN5.2	205	368	4.31
									TN5.3	737	140	2.65
TN6*	W	39	02:01	03:01	07:02	15:01	03:02	07:02	TN6.1		957	4.08
									TN6.2	2,064	487	5.47
TN7	W	22	02:01	03:05	07:02	07:02	07:02	07:02	TN7.1		475	3.25
									TN7.2	163	510	2.66
TN8	W	50	02:05	30:02	18:01	51:01	05:01	15:02	TN8.1		972	3.10
									TN8.2	161	720	3.87
									TN8.3	468	550	4.84
TN9	AA	21	02:01	26:01	40:01	57:01	03:04	18:01	TN9.1		1,353	3.52
									TN9.2	570	1,374	3.28
									TN9.3	2,346	1,736	2.63
TN10	W	38	02:01	02:01	27:05	44:02	01:02	05:01	TN10.1		910	5.00
									TN10.2	217	602	4.26
									TN10.3	326	766	4.32
TN11	AA	22	23:01	23:01	44:03	58:01	04:01	07:01	TN11.1		471	4.78
									TN11.2	337	518	4.42

W, White; AA, African American;

*Subject TN6 initiated Atripla treatment 37 days prior to final sample collection.

### Viral adaptations contribute significantly to alterations in Gag and Nef diversity

We next sought to determine the proportion of the observed amino acid variability of HIV that corresponds to adaptations. Using a list of known HLA-associated HIV adaptations, which have been statistically determined from large cohort studies [42, 43] and largely confirmed with functional cellular assays [44–46], we calculated the proportion of polymorphic amino acid sites subject to HLA-associated change within Gag, Pol and Nef. Across all three proteins examined, a median 25% (range, 0–75%) of all polymorphic sites mapped to known locations of adaptation. Stratification by protein identified that Gag and Nef exhibited similar levels of HLA-associated change, with a median of 50% (range, 0–100%) and 48% (range, 0–100%), respectively, across all subjects within the cohort (Fig 2D). Pol, however, exhibited significantly less change with a median of 0% (range, 0–10%) across all subjects.

### HLA-associated HIV adaptations can occur early in infection and are retained throughout disease progression

**Fig 3:**
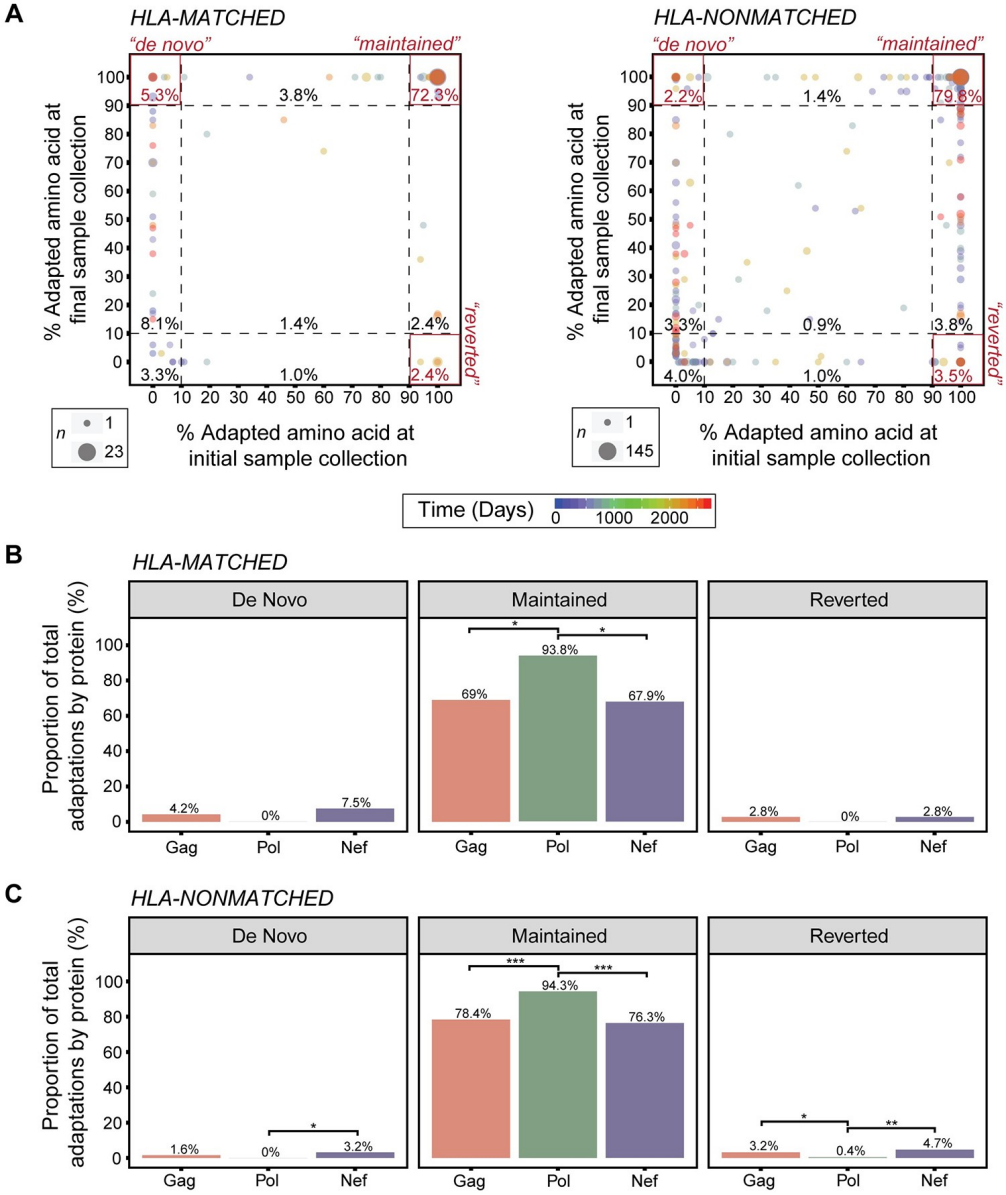
High proportion of viral adaptations are present early and retained over time. **A)** Adaptation dynamics between initial and final sample collection suggests that most HLA-matched adaptations (adaptations associated with host HLA class I alleles; 209 total adaptations) and HLA-nonmatched adaptations (adaptations not associated with host HLA class I alleles; 1702 total adaptations) are retained over time at ≥ 90% frequency in the viral quasispecies. Each point reflects a single HLA-associated HIV-1 adaptation. Size of each point displays the degree of superimposition of points. Color reflects the time in days between initial and final sample collection. Percentages indicate the proportion of total HIV-1 adaptations located within each quadrant. **B)** Comparison between proteins for the proportion of *de novo*, *maintained* and *reverted* HLA-matched adaptations over time (71, 32 and 106 adaptations examined for Gag, Pol and Nef, respectively). A significantly greater proportion of *maintained* adaptations in Pol was identified, compared to Gag (*, *p* = 0.025) and Nef (*, *p* = 0.022). However, no difference in the proportion of *de novo* or *reverted* adaptations between proteins was identified. **C)** HLA-nonmatched adaptations were also compared by protein (498, 281 and 923 adaptations examined for Gag, Pol and Nef, respectively). In this category, Pol exhibited a significantly reduced proportion of *de novo* adaptations compared to Gag (*, *p* = 0.013) and *reverted* adaptations compared to Gag (*, *p* = 0.036) and Nef (**, *p* = 0.004). However, Pol exhibited a significantly greater proportion of *maintained* adaptations compared to Gag (***, *p* < 0.001) and Nef (***, *p* < 0.001). Analyses in (B) and (C) using three-sample test for equality of proportions and Holm-corrected pairwise comparison of proportions.

Deep sequencing of HIV quasispecies from subject WA9 and subjects within the TN cohort were combined to assess viral adaptation dynamics over time (S3 and S4 Figs). Of particular interest were: (1) *de novo* adaptations, defined as being at < 10% frequency at initial sample collection and ≥ 90% frequency at final sample collection; (2) *maintained* adaptations, defined as being at ≥ 90% frequency at both initial and final sample collection; and (3) *reverted* adaptations, defined as being at ≥ 90% frequency at initial collection and < 10% frequency at final collection (Fig 3A). Here, we expected to observe an increase (or accumulation) in HLA-matched adaptations (associated with an HLA allele in the subject’s HLA class I repertoire) in response to host HLA-associated CTL immune pressure, and reduction (or reversion) in HLA-nonmatched adaptations with no relevance to the host’s HLA repertoire (likely present in the founder/transmitted virus).

Across all subjects, 72% of HLA-matched and a significantly higher proportion (79%) of HLA-nonmatched adaptations (*X*^*2*^[1] = 6.032, *p* = 0.014; two-sample test for equality of proportions) in these subjects were *maintained* at ≥ 90% frequency in the viral quasispecies between initial and final sample collection (Fig 3A). The high level of maintenance over time in both categories suggests that many HLA-associated HIV adaptations harbor minimal fitness cost and/or extensive compensatory mutations that offset the cost of retaining these adaptations by restoring efficient viral replication [10].

No statistical difference between the proportion of total HLA-matched and HLA-nonmatched adaptations in the *reverted* category was detected (*X*^*2*^[21] = 0.423, *p* = 0.515; two-sample test for equality of proportions), encompassing approximately 2% of all HLA-matched and 3% of all HLA-nonmatched adaptations present in these subjects. This suggests that overall, only a small fraction of HLA-associated HIV adaptations revert in this cohort (Fig 3A). Notably, of these reversions (S2 Table), a subset are located within or flanking epitopes eliciting responses in the acute WA cohort (GP**G**HKARVL, KEKGGLEGLI**H**, **R**PQVPLRPMTY, RPMTYKAA**V**, YTPGPG**I**RY, AFHH**M**AREL, FLKE**Q**GGL), which indicates these sites are likely under strong *in vivo* immune pressure. Moreover, of the HLA-nonmatched adaptations undergoing *reversion*, a subset of these overlap with *reversions* seen in another geographically and demographically distinct cohort [13], suggesting these sites of adaptation with predicted fitness cost have not been subject to compensatory fixation (S3 Table). Three of these adaptations correspond to sites within epitopes eliciting responses in the acute WA cohort (GP**G**HKARVL, YTPGPG**I**RY, RPMTYKAA**V**).

Across all subjects, a greater proportion of HLA-matched (5%) than HLA-nonmatched (2%) adaptations arose *de novo* and increased in frequency to fixation or near-fixation (≥ 90% frequency) in the viral quasispecies over time (*X*^*2*^[1] = 5.683, *p* = 0.017; two-sample test for equality of proportions; Fig 3A). Notably, the accumulation seen in HLA-nonmatched adaptation in this cohort is, in part, due to an overlap with HLA-matched adaptations, such that 40% of *de novo* HLA-nonmatched adaptations coincidentally correspond with HLA-matched adaptations. Overall, levels of *de novo* HIV adaptation in the HLA-matched group indicate persistent HLA-associated immune pressure over time in this cohort (Fig 3A). Moreover, 16 adaptations were present at < 10% frequency at initial sampling but did not reach the 90% frequency threshold at final sampling and likely indicate late/delayed viral adaptations (S4 Table). Of these *de novo* adaptations, three (FLKE**E**GGL, TPGPG**V**RYPL, KEKGGLEGL**I**) are contained within epitopes eliciting responses in the acute WA cohort. It should be noted that a comparison of HLA-matched and HLA-nonmatched adaptation in the viral quasispecies of the initial sample for each subject showed a 1.3-fold higher level of HLA-matched adaptation (median 26%; range 13–47%) versus HLA-nonmatched adaptation (median 20%; range 19–30%), supporting additional early viral adaptations not captured in this analysis (*p* = 0.034; Paired Wilcoxon test; S3 Fig).

In comparisons between proteins, Pol had approximately 24% and 25% more *maintained* HLA-matched adaptations than Gag and Nef, respectively, with approximately 93% of all HLA-matched adaptations in Pol in the *maintained* category. Similarly, Gag and Nef harbored most (approximately 69% and 67%, respectively) of their HLA-matched adaptations in the *maintained* category, emphasizing that across all proteins analyzed, most adaptations initially present in the subject viral quasispecies are *maintained* over time (Fig 3B). A similar scenario was seen in the HLA-nonmatched category, whereby Pol exhibited the greatest proportion of *maintained* HIV adaptations, having approximately 15% and 18% more than Gag and Nef, respectively (Fig 3C). Yet, all proteins displayed a high degree of retention of HLA-nonmatched adaptations over time, with approximately 78%, 94% and 76% of all HLA-nonmatched adaptations in Gag, Pol and Nef being *maintained*, respectively (S6 and S7 Figs). These results highlight the propensity for HIV to retain adaptations over time in both selective and non-selective immune environments.

### Accumulation of adaptation in Gag, Pol and Nef is predominantly associated with HLA-B and -C mediated selection pressure

**Fig 4:**
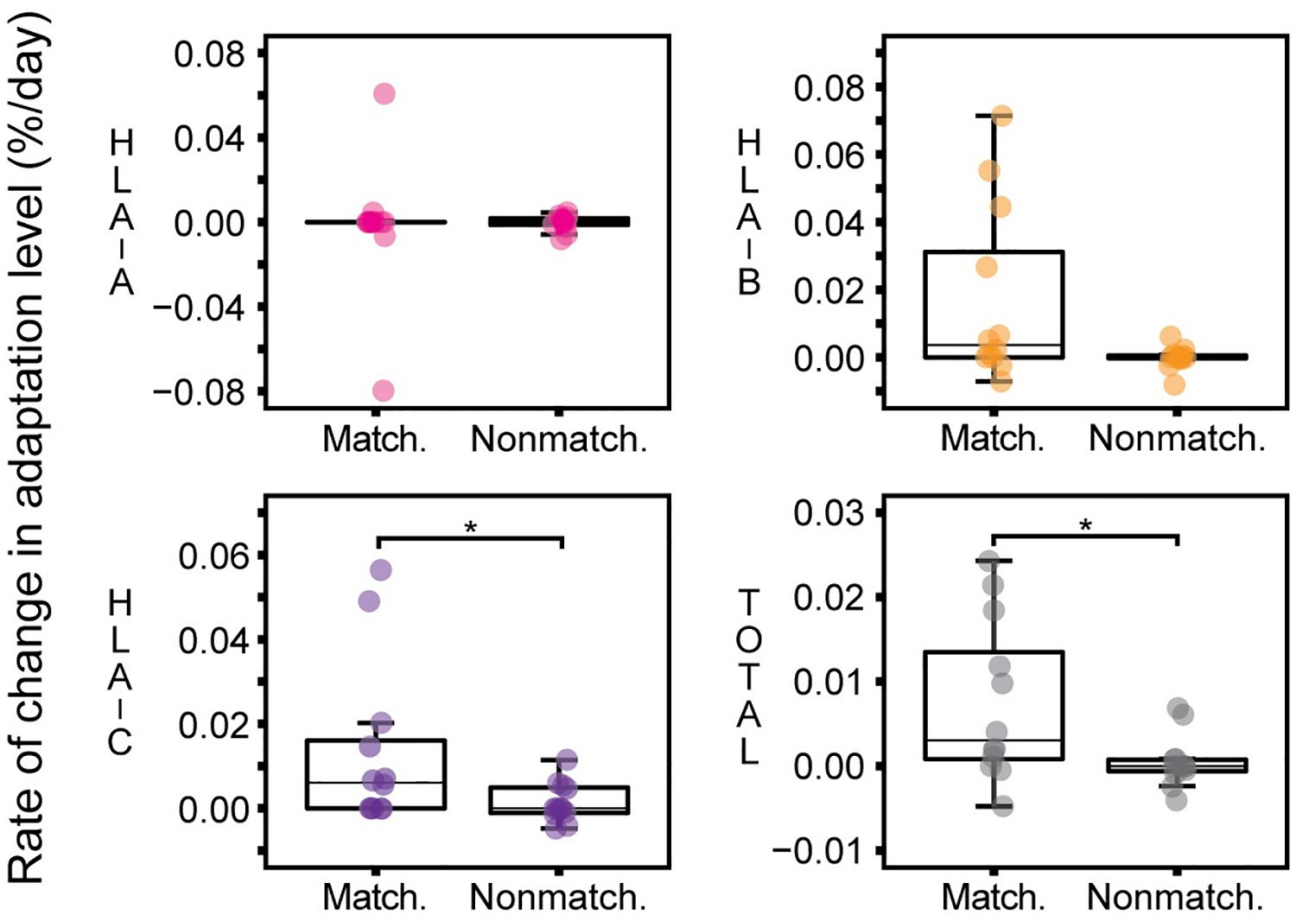
Accumulation of HLA–C-associated HLA-matched adaptations is suggestive of strong HLA–C-associated CTL immune pressure. HLA-C exhibits the greatest difference between HLA-matched and HLA-nonmatched adaptation rate (*, *p* = 0.029), and appears to greatly contribute to the difference in total HLA-matched and HLA-nonmatched adaptation rate (*, *p* = 0.037). The HLA-matched and HLA-nonmatched rate of change does not differ significantly for HLA-A (*p* = 0.689), however a trend was identified for HLA-B (*p* = 0.068). Analyses using paired Wilcoxon test.

We next examined the rate of change in *de novo* adaptations, with respect to the restricting HLA class I molecule, to identify differences in CTL selection pressure associated with the three HLA class I loci. For this analysis, HLA-nonmatched adaptations acted as a control for the level of background variability. The HLA-matched and -nonmatched rate of change did not differ for HLA-A (*p* = 0.689; paired Wilcoxon test) (Fig 4). We identified a trend of accumulating HLA-matched adaptation for HLA-B over time (*p* = 0.068), and a significantly higher average rate of change of HLA-matched adaptation for HLA-C (*p* = 0.029). Overall, these data indicate that the overall increase in total HLA-matched adaptation (*p* = 0.037) largely comprises accumulation of HLA-B- and HLA-C-associated adaptations, rather than those related to HLA-A in this cohort. We were unable to identify any correlations between increased HLA-matched or–nonmatched adaptations and viral load or CD4^+^ T cell count (S8 Fig).

### Accumulation of HLA-matched adaptations, combined with maintained HLA-nonmatched adaptations, are likely to increase viral adaptation in circulating HIV strains

**Fig 5:**
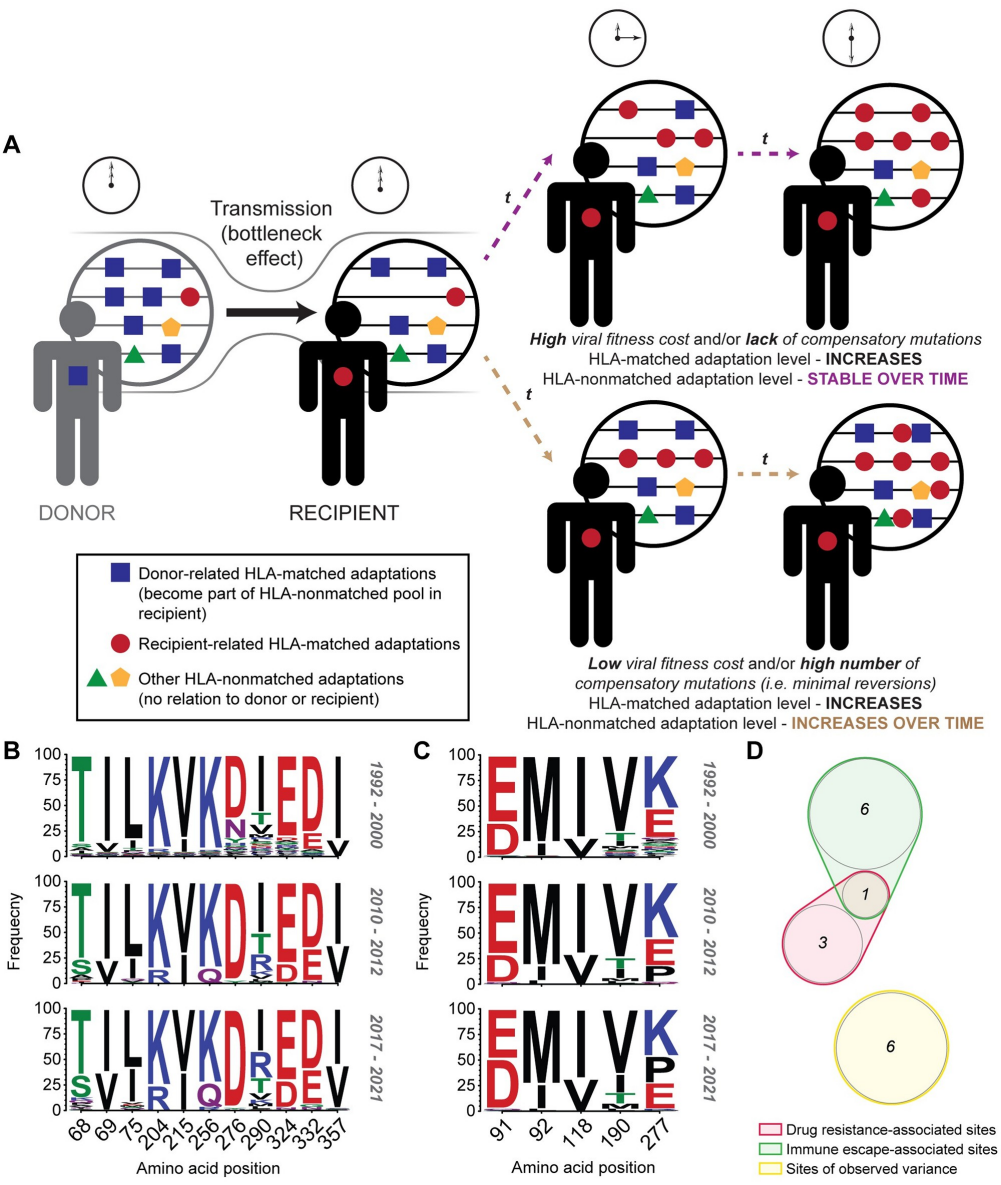
Evidence of accumulation of viral adaptations at a population level. **A)** Schematic of viral adaptation dynamics following transmission between HLA-mismatched donor-recipient pairs. Viral adaptations can be separated into two types: HLA-matched (adaptations relevant to the host’s HLA alleles; red circle) or HLA-nonmatched (adaptation to all HLA alleles, excluding those of the host; green triangle, yellow pentagon). HLA-matched adaptations have been shown to increase over time within an ART-naïve recipient, as a result of HLA-associated CTL immune pressure. Alternatively two potential scenarios related to HLA-nonmatched adaptations have been proposed, one of maintenance (where population-level HLA-nonmatched adaptations are maintained low over time due to high viral fitness costs and/or low numbers of compensatory mutations), or increasing adaptation (where population-level HLA-nonmatched adaptation steadily increase over time due to low viral fitness cost and/or high numbers of compensatory mutations facilitating maintenance of donor-relevant adaptations; blue square). **B & C)** Clade B sequence comparison of the Pol gene spanning approximately 30 years in Western Australia highlights key amino acid changes likely due to the effect of maintained HLA-nonmatched adaptations. Available *protease* and *reverse transcriptase* sequences obtained from ART-naïve subjects as part of clinical follow-up from 1992–2000 (*n* = 131), 2010–2012 (*n* = 147) and 2017–2021 (*n* = 80) were compared to identify amino acid differences between cohorts. Sites in (B) show those that showed persistent change over the approximate 30 year time period. Sites in (C) highlight those exhibiting significant change only in 2017–2021. Site numbering is with reference to HXB2. Significant change was determined using false discovery rate-corrected Fisher’s exact test. **C)** Stratification of the 16 identified sites in (B) and (C) suggests that drug resistance and immune escape variants increase in frequency over time in the Western Australian population.

In this set of subjects (WA9 and TN cohort), we identified individuals harboring high levels of HLA-nonmatched adaptation, in addition to accumulating HLA-B and -C-associated HLA-matched adaptations over time. These data suggest that viral adaptations can be readily transmitted and often retained at high frequencies in the viral quasispecies following transmission (Fig 5A). Moreover, these findings support recent studies identifying similar phenomena at the population level [17–20].

To further explore the likelihood of increasing viral adaptations within populations, we obtained pre-ART Pol sequences from the plasma of HIV-infected subjects spanning approximately 30 years in the Western Australian population. We examined clade B sequences from 1992–2000 (*n* = 131), 2010–2012 (*n* = 147) and 2017–2021 (*n* = 80) to identify differences in amino acid frequencies between cohorts (S5 and S6 Tables). Eleven sites undergoing consistent change across the three cohorts were identified using false discovery rate-corrected Fisher’s Exact test (Fig 5B). A further five sites showed a changing trend in 2010–2012 that became significantly different by 2017–2021 (Fig 5C). Of these 16 sites, 25% (4/16) were sites under known drug-imposed selection, 44% (7/16) were sites under known HLA-associated CTL immune pressure, of which one site was associated with both HLA- and drug-associated selective pressures (Fig 5D). Similar results were obtained when we utilized clade B proviral Pol sequences from the original study on HIV adaptation in circulating strains (*n* = 427) in Western Australia between 1995–2002 [7] (S7 and S8 Tables).

Most (6/7) of the sites under HLA-associated immune pressure are related to HLA-B-associated selection pressures (T68X, HLA-B*52:01; E91D, HLA-B*44 [47]; I290T/R, HLA-B*51 [17]; E324D, HLA-B*07:02; D332E, HLA-B*35:01 [7]; I357V, HLA-B*40:01; and V190I, HLA-A*02 [48]). All of these HLA alleles have remained common in Western Australia over the past 30 years, suggestive of accumulating viral adaptation at a population level. Of the seven sites detected, position 91 represents the scenario whereby the non-consensus amino acid (D, aspartic acid) transitions over time to reach the same frequency as the consensus amino acid (32% in 1992–2000; 28% in 2010–2012; 50% in 2017–2021). The emergence of this viral adaptation over time is supported by allele frequency data in Western Australia (allelefrequencies.net), which estimates that the associated HLA-B*44 allele is carried by approximately 25% of the population. Moreover, given the similar trend seen at position 190, 324, 332 and 357, in combination with the approximate 13–20% HLA allele carriage rate in the population of Western Australia, we predict these sites may transition such that the viral adaptations become the consensus amino acid in the circulating virus. To further support this prediction, recent work conducted in Italy spanning 2003–2016 identified seven sites associated with viral adaptation of increasing frequency, of which three overlap with those identified in this study (V190I, D332E and I357V) [49]. Notably, the HLA alleles associated with these viral adaptations are also well represented in the Italian population, supporting the notion of HLA-driven HIV evolution at a population level.

## Discussion

Given global efforts towards early diagnosis and immediate or rapid ART initiation [50], studies examining *in vivo* HIV evolution in the absence of ART have become challenging [51–53]. This study has sought to utilize three well characterized clinical cohorts sampled at distinct phases of HIV infection to improve our understanding of HIV adaptation to CTL-mediated immunity.

The acute WA cohort provides supporting evidence for the previously identified immunogenicity of the Gag and Nef proteins (and to a lesser extent, Pol), and their preferential targeting by T cells [32–34]. Rather than utilizing protein-specific peptide pools, this study used single peptides representing known clade-B-based HLA-specific HIV epitopes relevant to the HLA class I repertoire of each subject. Using this individualized approach, we were able to identify numerous responsive peptides, with many overlapping between subjects with differing clades (S1 Table), suggesting that interclade variation does not always impact CTL-mediated immunity [54]. Although this work confirms previous findings by others, these peptides are largely from the Gag, Pol and Nef proteins (20.8%, 34.3% and 24.8%, respectively), and as such may skew the proportion of protein responses seen in this study. We may have detected more responses with clade-specific or autologous virus-specific peptides. Nevertheless, strong HIV-specific CTL responses prior to seroconversion were uncovered in keeping with early selection pressure on the virus (S1 Fig). Emerging viral adaptations within epitopes corresponding to tested peptides in the ELISpot assay suggests that although CTL-mediated immunity can contribute to the control of HIV infection as previously reported [1, 2, 55], this also provides an environment for the selection of viral adaptations, which may be seeded into the proviral reservoir [27–30].

The next-generation sequencing data from the TN cohort and subject WA9 (WA cohort) allowed for high throughput examination of the *in vivo* evolutionary dynamics of the Gag, Pol and Nef proteins over time, which were determined in the acute WA cohort as key targets of the CTL response. These data support previous literature identifying the highly conserved nature of Pol [13, 56]. In this study, Gag and Nef exhibited higher levels of genetic variation, and viral adaptations largely accounted for the variation seen in these proteins. However, it is important to note that a degree of this genetic variation may relate to flexible residues which coincidentally match known viral adaptations. Furthermore, in this cohort, Gag and Nef, exhibited accumulation of *de novo* HLA-matched adaptations over time, particularly those associated with HLA-B and -C. Interestingly, despite the established dominant influence of HLA-B in driving overall HIV evolution [57], these data support that HLA-C may also have an important role in driving evolution in the Gag and Nef proteins.

Utilizing the same deep sequencing data, we had the capacity to explore the high-resolution dynamics of HLA-nonmatched adaptations. These HLA-nonmatched adaptations constitute those viral adaptations likely inherited from the donor derived founder virus with no adaptive advantage in the HLA environment of the recipient. Although this study is limited by not knowing donor-related HLA class I genotype, the list of known HLA-associated HIV adaptations [42, 43] was used to identify HLA-nonmatched adaptations undergoing reversion (S2 Table). A subset of these adaptations were predicted to be of high fitness cost given their reversion in a geographically and demographically distinct cohort (S3 Table), and therefore suggestive of HLA-mismatched donor-recipient HIV transmission. Furthermore, despite being unable to conduct functional T cell assays on these subjects due to the historical nature of this cohort and unavailable PBMCs, a portion of these reverting adaptations corresponded to sites within epitopes eliciting responses in the acute WA cohort, and suggests that these sites may be under strong *in vivo* immune pressure. Interestingly, we identified fewer HLA-nonmatched reverting adaptations than predicted [43], potentially due to the timing of initial sampling of the subjects in this cohort (i.e. initial sample collected within the first year of infection). Alternatively, given reversion is largely dependent on both the cost of the adaptation on viral replication and conferred survival advantage against HLA-associated CTL selection pressures, the presence of linked compensatory mutations offsetting the cost of maintenance is also possible. To date, compensatory mutations remain understudied, and as such, we were unable to detect the extent of compensatory mutations involved in the maintenance of HLA-nonmatched adaptations in this cohort. We identified a substantial number of HLA-nonmatched adaptations present upon initial sampling and maintained over time, which we speculate may be due to extensive pre-adaptation in the founder virus, and may involve numerous linked compensatory mutations. These data combined with our previous work [13] suggests fixation of viral adaptation at the individual and population level involves a complex network of primary and secondary/compensatory mutations affecting viral fitness, rather than isolated single changes with large fitness impact.

Using the same data set, we were also able to identify *de novo* HLA-matched adaptation, which likely relates to sites of strong selective pressure. Some of these sites were found to be within epitopes eliciting IFNγ responses in subjects from the acute WA cohort, and when combined with previous functional data [45, 46], further suggests that they may be under strong *in vivo* immune pressure. Moreover, we also identified numerous HLA-matched adaptations that increased in frequency over time but did not reach the “fixation” threshold of >90% at the final sampling time point. The latter adaptations we predict may be under low/moderate HLA-associated CTL selective pressure (S4 Fig) resulting in delayed escape, which in the absence of longitudinal sampling would be difficult to identify. Furthermore, these sites exhibiting delayed escape may relate to regions harboring subdominant epitopes, or alternatively, sites subject to significant structural/functional constraint. Identification of the latter is likely to be of significant interest for immunogen design.

To corroborate our findings and examine if viral adaptations may indeed be increasing in frequency in the circulating HIV strains within a population, we examined cross-sectional data obtained from Western Australia spanning approximately 30 years. Of the seven positions detected as being viral adaptation sites and increasing in frequency, E91D highlights the capacity for a viral adaptation to reach the same frequency as the consensus amino acid within a population over time. This phenomenon subverts the idea that a population consensus strain is equivalent to ‘wildtype” virus. Evidence of fixation of viral adaptations at the population level has been presented in various studies [7, 17–21] that indeed overlap with specific sites identified in this study, but the degree to which this impacts the fitness of transmitting viruses or abrogates the efficacy of immunogens based on population consensus sequences is inherently difficult to characterize.

Studies of *in vivo* viral evolutionary dynamics can help identify specific changes in residues that appear to favor the virus rather than the host. It has been long proposed that such changes may abrogate effective vaccine or cure approaches. This study provides information about early changes in potential regions of interest by identifying sites of reversion (particularly those overlapping with other studies or cohorts) and sites of delayed immune escape. Moreover, in the context of therapeutic vaccine development, these findings provide a guide to the potential level of viral adaptation harbored in the latent reservoir. Notably, in individuals diagnosed earlier in the epidemic and withheld ART for some time or individuals with poor ART adherence, we predict that the level of adaptation is likely to be significant, particularly in the proteins Gag and Nef [30]. If therapeutic vaccine development for the elimination of HIV aims to make use of cellular immunity, adapted HIV variants would need to be considered. For example, inclusion of epitopes where the mutational barrier to escape is predicted to be high and of significant replicative importance (S2 and S3 Tables) may improve HIV control. Alternatively, avoiding regions with common escape variants may reduce immune priming towards subdominant epitopes or immune exhaustion [37]. Ultimately, a greater understanding of early viral adaptation will elucidate patterns of evolution at the population level and enhance approaches for preventative and therapeutic vaccine development.

## Materials and methods

### Ethics statement

All subjects gave written and verbal informed consent prior to participation and samples were anonymized. Institutional review board (IRB) approval for sample collection was obtained prior to the commencement of the study by the Vanderbilt Institutional Review Board (IRB100061; IRB030005). Reciprocal human ethics approval was obtained from the University of Western Australia (RA/4/20/4583) and Murdoch University (2017/242).

### Subjects–WA cohort

Eleven subjects with acute and early HIV infection were recruited from outpatient clinics and/or hospital admissions (Royal Perth Hospital and Sir Charles Gairdner Hospital, Perth, WA, Australia). The majority of subjects were male (9/11), with a median age of 37 (range 18–47) years. Six subjects had clade B, three subjects had clade AE, one subject had clade C and one subject had clade AG HIV infection. Five subjects had known dates of HIV transmission and conservative estimates of inferred transmission dates were generated for the remaining six subjects based on mean cumulative duration of Fiebig staging (31 days for Fiebig IV [*n* = 5 subjects]; 101 days for Fiebig VI [*n* = 1 subject]), as reported by Cohen and colleagues [58]. Initial viral load, CD4^+^ T cell count and clinical history were available for the majority of subjects (7/11). Two subjects presented pre-seroconversion with positive p24 antigen and the absence of any antibody, indicating Fiebig stage II. Seven subjects presented with indeterminate Group 4 western blot, indicating Fiebig stage IV. One subject presented with a fully positive western blot, indicative of Fiebig stage VI, and had reported seroconversion-like symptoms three months earlier, suggestive of recent acute HIV infection. One subject presented with an indeterminate Group 3 western blot, indicative of Fiebig stage III, with an apparent delayed seroconversion illness. This subject had commenced PEP (atazanavir and ritonavir) three months earlier, two days after an HIV exposure event, the second of two HIV exposures within 20 days. PBMCs and plasma were separated and stored for each subject. Eight subjects had one sample collected, two subjects had two samples collected and one subject had seven samples collected. The first samples were collected from each subject a median of 46 (range 27–177) days post HIV transmission. PBMCs and plasma samples were collected from the Fiebig stage VI subject 16 days after presentation (117 days post HIV transmission) and from the Fiebig stage III subject, with apparent delayed seroconversion after PEP, approximately 177 days after first HIV exposure (Table 1).

### Subjects—TN cohort

Eleven subjects with early-chronic HIV infection were recruited and followed longitudinally from the Vanderbilt-affiliated Comprehensive Care Center (Nashville, TN, United States). All subjects were male, with a median age of 37 (range 20–50) years at earliest sample collection, and infected with HIV clade B. Eight subjects had known month and year of most recent negative HIV serology, and conservative estimates of time since last negative HIV test were generated. For example, HIV negative serology dated at October 2002 (estimated to be 1^st^ October 2002) on a sample collected on 18^th^ November 2003, was inferred to be 413 days since last negative HIV test. For the eight subjects, the median time since last negative HIV test was 357 (range 152–975) days. Viral load and CD4^+^ T cell count were available, with all eleven subjects exhibiting a relatively high median CD4^+^ T cell count of 731 (range 432–1,353) cells/mm^3^ and low median plasma viral load of 3,288 (range 162–100,000) HIV RNA copies/mL at initial sample collection. PBMCs and plasma was separated and stored for each subject, however only plasma was accessible for this study. The eleven subjects had a median of 3 (range 2–4) time points collected, with the time between the initial and final time points being a median of 468 (range 163–2,676) days. All subjects were ART naïve at initial sample collection and continued to be for the duration of time point collection, except for subject TN6, who initiated Atripla (efavirenz, emtricitabine, and tenofovir disoproxil fumarate) treatment 37 days prior to final sample collection.

### IFNγ ELISpot assay on cryopreserved PBMC samples

HIV-specific HLA class I-associated CTL responses in the WA cohort were evaluated by IFNγ ELISpot assay. HIV peptides were selected based on subject HLA class I alleles and sufficient PBMC available, with a median of 56 (range 35–71) peptides tested per subject. Cryopreserved PBMCs were thawed, resuspended in culture medium (10% FCS/RPMI-1640) and left to settle overnight at 37°C. Viable cells were enumerated by trypan blue exclusion using a Neubauer haemocytometer. 100,000 cells were dispensed per well in duplicate with HIV peptides (5 μg/mL final concentration), anti-CD3 antibody, CEF antigens (Mabtech, Upsala, Sweden) or culture media alone at a final volume of 150 μL/well. IFNγ ELISpot assays were performed as previously described [36]. Briefly, nitrocellulose-backed 96 well Millipore plates (Bedford, MA, United States) were coated overnight at 4°C with 2 μg/mL of anti-IFNγ antibody. Plates were washed and blocked with culture medium (RPMI-1604 supplemented with 10% fetal calf serum) for 30 minutes minimum at room temperature, after which cells and stimulants were dispensed for overnight incubation at 37°C. Plates were washed with sterile PBS and biotinylated IFNγ antibody added (100 μL/well, Mabtech, Victoria, Australia) for 2 hours at room temperature, after which plates were washed and streptavidin horseradish peroxidase (Mabtech, Victoria, Australia) was added for 1 hour at room temperature. Plates were washed and developed with Tetramethylbenzadene substrate (100 μL/well, Mabtech, Victoria, Australia) for 10 minutes at room temperature. Plates were washed extensively with Milli-Q H_2_O and left to dry prior to analysis on the AID iSpot Reader (AID, Strassburg, Germany) with AID software (5.0 B7337). IFNγ count settings were based on spot size (>50), intensity (> 25) and gradient (>5) as recommended by AID. Responses were determined by subtracting the mean of unstimulated wells from the mean of antigen-stimulated wells. Results are presented as spot forming units (SFU) per million cells. Responses were considered positive if they were ≥ 50 SFU after background subtraction. Wells with spot numbers classified as too numerous to count (TNTC) were awarded a spot count of 400 and reported as 4000 SFU/10^6^ cells. The median response detected for cells alone, with no stimulus added, across 51 assays was 20 (range 3–78) SFU/10^6^ cells. For the positive controls, PBMC from all subjects responded to anti-CD3 stimulation (TNTC), while 10/11 subjects demonstrated responses to CEF antigen (1,755 median, range 162–4000 SFU/10^6^ cells, *n* = 10).

### HIV peptides

HIV peptides were synthesized by Invitrogen (Melbourne, Australia). Peptides were reconstituted in dimethylsulphoxide 10 mg/mL and stored at -80°C. Peptide stock solutions at 50 μg/mL were diluted 1:10 for use in ELISpot assays.

### HLA class I genotyping—WA cohort

Low resolution HLA class I genotyping was performed by the Department of Clinical Immunology at Royal Perth Hospital (Perth, WA, Australia), as previously described [59]. Briefly, genomic DNA was isolated from all subjects and PCR amplification of the HLA-A, -B and–C loci was conducted using sequence specific primers. Products were resolved to two-digit level resolution based on exon 2–3 sequences using standard sequence-based typing.

### HLA class I genotyping—TN cohort

High resolution HLA class I genotyping was performed at the Institute for Immunology and Infectious Diseases (IIID), Murdoch University (Perth, WA, Australia)—an American Society for Histocompatibility and Immunogenetics (ASHI) and National Association of Testing Authorities (NATA) accredited laboratory—as previously reported [13]. Briefly, genomic DNA was isolated from all subjects and underwent HLA class I locus-specific barcoded-PCR amplification. Amplified products were pooled in equimolar ratios by subject and sequenced on the Illumina MiSeq platform, with the output quality-filtered and resolved to four-digit level resolution using the IMGT HLA allele database [60].

### Viral sequencing—WA cohort

HIV viral sequencing using a nested PCR approach was performed on subject plasma samples at IIID, Murdoch University (Perth, WA, Australia), as previously described [61]. Briefly, HIV RNA was extracted from subject plasma samples using the Life Technologies MagMAX Viral RNA Isolation Kit, as per the manufacturer’s instructions. Two overlapping RT-PCRs were performed to cover the entire HIV genome. Resultant first round products were used as templates in two separate nested PCRs targeting the Gag, Pol and Nef genes. Standard bulk sequencing was carried-out using the Roche 454 Life Sciences GS-FLX platform. PCR products were quantified and equimolar pooled for each individual. Products were ligated to adaptors and clonally amplified on capture beads in water-in-oil emulsion micro-reactors, with the enriched products being sequenced on picotitre plates. Nucleotide data was collected and quality filtered using the Roche 454 software (default settings). Viral sequences were aligned to the HXB2 reference HIV clade B strain (GenBank accession number K03455) and a consensus sequence was generated for each subject using an in-house developed alignment tool (http://www.iiid.com.au/software/vgas).

### Viral sequencing—TN cohort & longitudinal subject WA9

Deep HIV viral sequencing using a nested PCR approach was performed on subject plasma samples at IIID, Murdoch University (Perth, WA, Australia), as previously reported [13]. Briefly, HIV RNA was extracted from subject plasma samples using the Life Technologies MagMAX-96 Viral RNA Isolation Kit, as per the manufacturer’s instructions. Targeted RT-PCRs were performed to cover the Gag, Pol and Nef genes. Resultant first round products were amplified by nested PCR. Deep bulk sequencing was conducted on the Illumina MiSeq platform. Second round PCR amplicons were quantified, equimolar pooled and enzymatically fragmented for each individual. Raw sequencing reads were quality trimmed (default MiSeq settings) and aligned to the HXB2 reference HIV clade B strain (GenBank accession number K03455) using QIAGEN Bioinformatics’ CLCbio Genomics Workbench 11. Aligned sequencing files were exported in BAM format and imported into an in-house developed analysis tool (http://www.iiid.com.au/software/vgas), with amino acid frequencies, consensus and majority sequences exported using a 3% nucleotide cut off.

### Collection of circulating HIV sequences from Western Australia over time

The HIV-1 sequence data from 1992–2000 was primarily from plasma samples collected prior to ART commencement through the Immunology Clinic at Royal Perth Hospital (Perth WA, Australia). Briefly, HIV RNA was extracted from plasma samples using the MagMAX-96 Viral RNA Isolation kit and converted to cDNA using the SuperScript III One-Step RT-PCR System with Platinum Taq High Fidelty Kit (Life Technologies, Carlsbad, CA, USA) as per the manufacturer’s instructions. Viral amplicons were purified using Agencourt AMPure XP Kit (Beckman Coulter, Brea, CA, USA) and prepared for sequencing, either via sanger-based methods or on the Illumina MiSeq sequencer (Illumina, San Diego, CA, USA). Analysis of the sequences were performed using an in-house developed analysis tool (http://www.iiid.com.au/software/vgas).

For the 2010–2012 (*n* = 147) and 2017–2021 (*n* = 80) sequences, HIV nested Pol amplification and sanger sequencing was performed utilizing plasma samples collected through the Immunology Clinic at Royal Perth Hospital (Perth WA, Australia) as previously reported [62]. Briefly, HIV RNA was extracted using the QIAGEN QIAamp Viral RNA kit, as per the manufacturer’s instructions. The extracted RNA was converted to cDNA, followed by nested PCR. Pol amplicons were sequenced using ABI Prism Big Dye terminator chemistry, and electrophoresis was performed on the ABI 3730xl instrument. Analysis of Pol sequences was performed using the ASSIGN editing tool (Conexio Genomics). HIV sequences from 2010–2012 were downloaded from GenBank (accession numbers KT228338 to KT229359). The original proviral HIV sequences dated between 1995–2002 were obtained as previously described in [7] (GenBank accession numbers DQ409341 to DQ409804).

Viral sequences for all time points were aligned using the ’DECIPHER’ package in RStudio. HIV subtyping analysis was done using the REGA HIV subtyping tool [63] and LANL QC online tool (http://www.hiv.lanl.gov/content/sequence/QC/index.html). Following amino acid translation and confirmation of clade type, sequences were filtered to exclude non-B subtypes. Ambiguous codes with two potential amino acids were counted as 0.5 each, whereas codes with >2 potential amino acids were excluded from the analysis. All data were aligned to the HXB2 reference HIV clade B strain (GenBank accession number K03455).

### Phylogenetic analyses

To confirm longitudinal sampling within subjects and clade association for each subject, a phylogenetic tree was constructed using MEGA X [64]. The best model function was used to infer the most appropriate evolutionary analysis method. For the consensus sequences obtained from the WA cohort, the Maximum Likelihood method and Tamura-Nei model [65] with a discrete Gamma distribution (G parameter = 0.3715) was used. For the majority sequences obtained from the TN cohort, the Maximum Likelihood method and Hasegawa-Kishino-Yano model [66] with a discrete Gamma distribution (G parameter = 0.6596) and allowance of evolutionary invariation (I = 41.16% sites) was used. Trees were drawn to scale, with branch lengths measured in the number of substitutions per site. HIV subtypes were further confirmed using the REGA HIV subtyping tool [63].

### Genetic diversity analysis

Calculation of the genetic diversity between subject viral sequencing data was conducted in MEGA X [64]. The pairwise distances function was used to calculate the number of nucleotide differences between longitudinal protein sequences, using the conservative complete deletion of gaps/missing data approach. The number of nucleotide differences (synonymous, nonsynonymous and combined) were normalized based on protein sequencing length to generate a comparable measure of mutational capability. For statistical analyses between proteins over time, the cumulative mutational capability was normalized based on time in days between initial and final sample collection.

### Determination of viral adaptation

Viral adaptation level was calculated as previously reported [13]. Briefly, prior studies have identified statistical associations between specific HLA class I alleles and amino acid polymorphisms across the HIV genome representing sites of immune escape [7, 59]. These HLA-specific polymorphisms were compiled and compared with subject viral amino acid frequencies, in combination with subject HLA class I genotyping, to quantify the level of viral adaptation [42]. Adaptations were grouped into either HLA-matched (viral adaptations present in the viral quasispecies with a known immune escape association with host HLA alleles) or HLA-nonmatched (viral adaptations present in the viral quasispecies but not associated to the subject’s HLA class I alleles). The level of viral adaptation was calculated as the proportion of the number of adaptations present, to the number of adaptations possible.

### Statistical analyses

Comparison between proportions involved either a 2-sample or 3-sample test for equality of proportions, followed by Holm-corrected pairwise comparison of proportions. Three-way grouped comparisons utilized a Friedman test and Holm-corrected paired Wilcoxon test for subsequent multiple comparisons. Two-way comparisons employed a paired Wilcoxon test. Mixed effects linear model was used to incorporate multiple measures per subject and account for longitudinal subject sampling. Correlation analyses used Spearman’s rho. Cross-sectional comparison of Pol sequences from Western Australia was conducted using false discovery rate-corrected Fisher’s exact test. Statistical analyses were performed using R version 4.1.0 for PC, with significance threshold set at α = 0.05.

## Supporting information

S1 Table WA cohort ELISpot responses. (XLSX)

S2 Table Reverting HLA-nonmatched adaptations. (XLSX)

S3 Table HLA-nonmatched adaptations undergoing reversion that overlap with Currenti et al., 2019. (XLSX)

S4 Table De novo (< 10% initial, ≥ 90% final) and other (< 10% initial, 10% ≤ x < 90% final) HLA-matched adaptations. (XLSX)

S5 Table Amino acid frequency changes in Pol between 1992–2021 in Western Australia. (XLSX)

S6 Table Consensus & non-consensus amino acid frequency changes in Pol between 1992–2021 in Western Australia. (XLSX)

S7 Table Amino acid frequency changes in Pol between 1995–2021 in Western Australia using proviral cohort data published by Moore et al., 2002. (XLSX)

S8 Table Consensus & non-consensus amino acid frequency changes in Pol between 1995–2021 in Western Australia using proviral cohort data published by Moore et al., 2002. (XLSX)

S1 Fig Emergence of viral adaptation within the HLA-B*07-associated GL9 epitope in subject WA9. **A)** Similar ELISpot IFNγ response to stimulation with adapted and non-adapted peptide. **B)** Transition over time from serine (S) to glycine (G) in viral quasispecies within the GL9 epitope.
(TIF)

S2 Fig Similar pattern of synonymous and nonsynonymous variation in Gag, Pol and Nef. Synonymous **(A)** and nonsynonymous **(B)** nucleotide variation in Gag, Pol and Nef exhibit similar patterns over time. No statistical difference was identified between the rate of synonymous polymorphisms in Gag, Pol and Nef (A; *p* = 0.386; Friedman test). However, a significant difference in the rate of nonsynonymous polymorphism (B; *p* = 0.015; Friedman test) was identified, which was predominantly driven by a higher rate in Nef, compared to Pol (**, *p* = 0.015; Holm-corrected Wilcoxon test). A trend was present when comparing the rate between Gag and Pol (*p* = 0.054) and Nef (*p* = 0.054).
(TIF)

S3 Fig Viral adaptation dynamics over time by protein. **A)** HLA-matched adaptation dynamics. **B)** HLA-nonmatched adaptation dynamics.
(TIF)

S4 Fig Viral adaptation dynamics over time by HLA-association. **A)** HLA-matched adaptation dynamics. **B)** HLA-nonmatched adaptation dynamics.
(TIF)

S5 Fig Evidence for viral adaptation prior to initial sampling during acute/early infection. **A)** HLA-matched adaptation level of Gag (*, *p* = 0.012) and Nef (**, *p* = 0.004) is significantly higher than Pol and exhibits a high degree of variation between subjects. **B)** HLA-nonmatched adaptation is similarly significantly higher in Gag (**, *p* = 0.001) and Nef (**, *p* = 0.001), than Pol, and displays less inter-subject variability than HLA-matched adaptation. Moreover, Nef appears to have greater levels of adaptation compared to Gag (*, *p* = 0.005). **C)** No significant difference between HLA-matched adaptation level of HLA class I loci (*p* = 0.558). **D)** HLA-C has a significantly higher level of HLA-nonmatched adaptation in this cohort, compared to HLA-B (*, *p* = 0.017). Analyses using Friedman test and Holm-corrected Wilcoxon test.
(TIF)

S6 Fig Protein-level viral adaptation changes over time. **A)** Changes in HLA-matched adaptation level over time by subject. Total HLA-matched adaptation level exhibited a significant positive correlation with time (*p* = 0.025). Whereas no significant correlations with time were identified for subject Gag (*p* = 0.052), Pol (*p* = 0.525) or Nef (*p* = 0.093) HLA-matched adaptation changes. **B)** No correlations with time were identified for total (*p* = 0.403), Gag (*p* = 0.936), Pol (*p* = 0.967) or Nef (*p* = 0.174) HLA-nonmatched adaptation level. Analyses conducted using mixed effects linear regression.
(TIF)

S7 Fig HLA-level viral adaptation changes over time. **A)** Changes in HLA-matched adaptation level over time by subject. Total (*p* = 0.025), HLA-B (*p* = 0.037) and HLA-C (*p* = 0.002) associated HLA-matched adaptation level exhibited a significant positive correlation with time. However, no significant correlation with time was identified for HLA-A (*p* = 0.600). **B)** No correlations with time were identified for total (*p* = 0.403), HLA-A (*p* = 0.531), HLA-B (*p* = 0.626) or HLA-C (*p* = 0.836) HLA-nonmatched adaptation level. Analyses conducted using mixed effects linear regression.
(TIF)

S8 Fig No correlation between change in HLA-matched adaptation level and viral load or CD4^+^ T cell count. Analyses using spearman’s rank correlation rho.
(TIF)

S9 Fig Comparison between HLA-association of viral adaptations for the proportion of *de novo*, *maintained* and *reverted* adaptations over time. **A)** A significantly lower proportion of HLA-B-associated *maintained* adaptations were identified, compared to HLA-A-associated adaptations (*, *p* = 0.024), and a similar trend was identified when compared with HLA-C-associated *maintained* adaptations (*p* = 0.052). However, no difference in the proportion of *de novo* or *reverted* adaptations between HLA-associations was found. **B)** No difference in proportions was identified between HLA-associations in the HLA-nonmatched category. Analyses conducted using three-sample test for equality of proportions and Holm-corrected pairwise comparison of proportions.
(TIF)
