# Supplementary figures and images for: Adaptation to HLA-associated immune pressure over the course of HIV infection and in circulating HIV-1 strains
Source: PLoS Pathog. 2022 Dec 16;18(12):e1010965. doi: 10.1371/journal.ppat.1010965 (PMC9803285; doi:10.1371/journal.ppat.1010965)

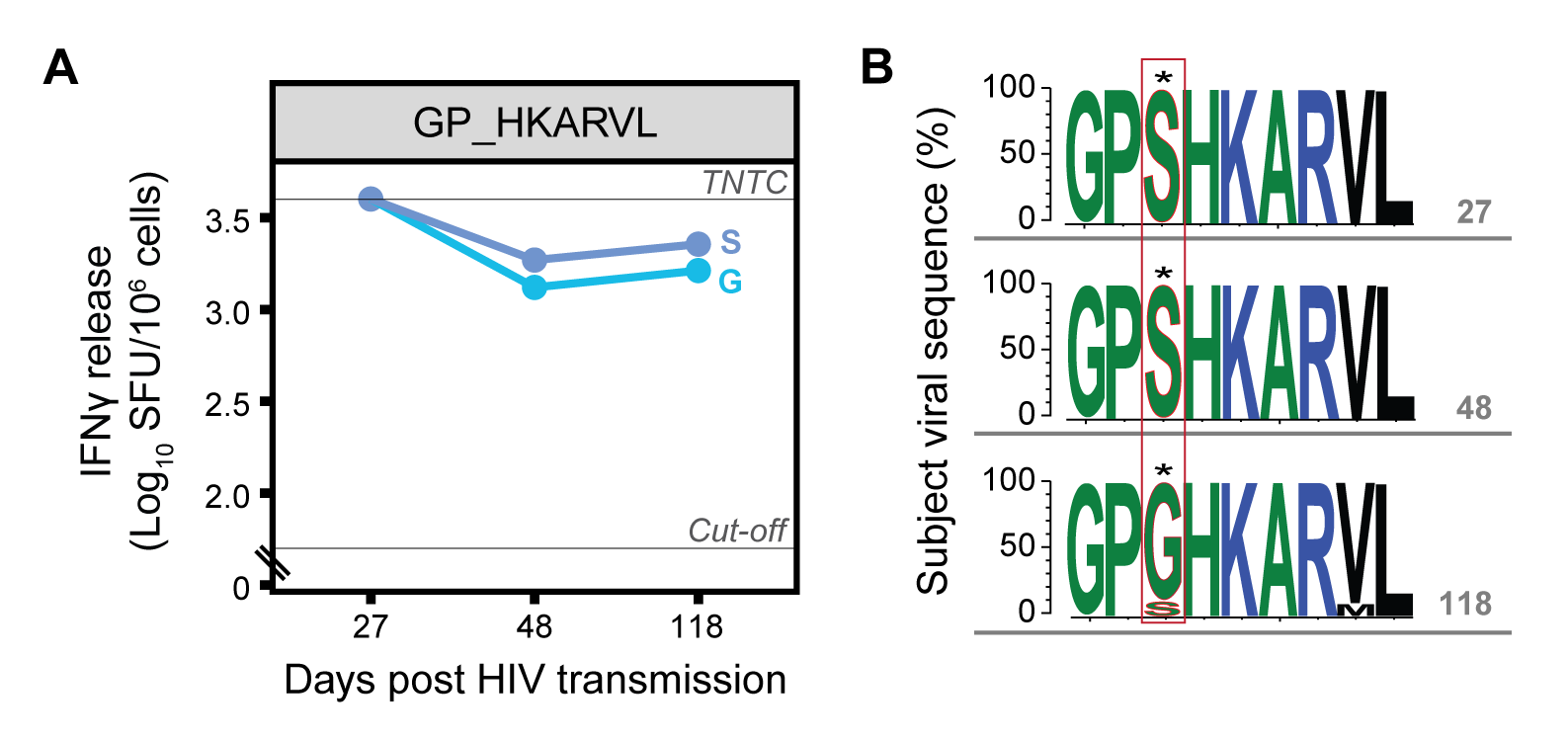

Supplement: S1 Fig — A) Similar ELISpot IFNγ response to stimulation with adapted and non-adapted peptide. B) Transition over time from serine (S) to glycine (G) in viral quasispecies within the GL9 epitope. (TIF) [file ppat.1010965.s009.tif]

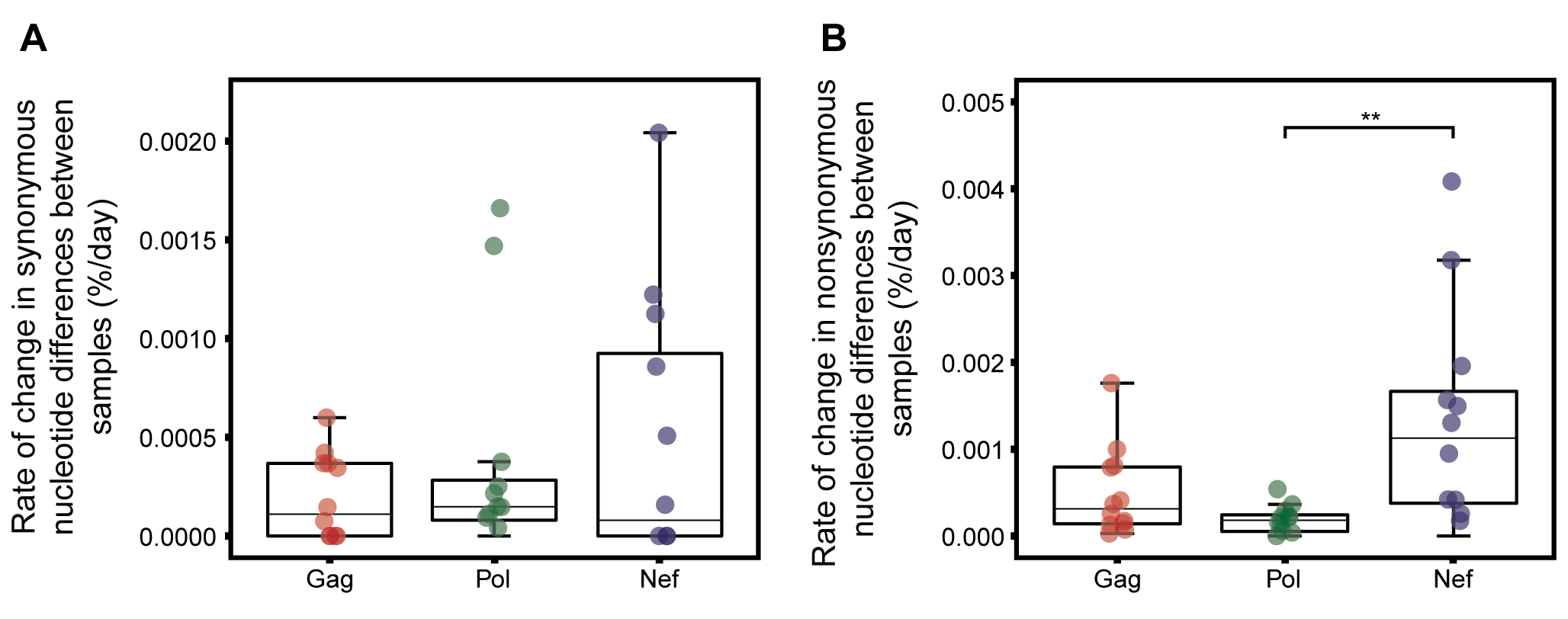

Supplement: S2 Fig — Synonymous (A) and nonsynonymous (B) nucleotide variation in Gag, Pol and Nef exhibit similar patterns over time. No statistical difference was identified between the rate of synonymous polymorphisms in Gag, Pol and Nef (A; p = 0.386; Friedman test). However, a significant difference in the rate of nonsynonymous polymorphism (B; p = 0.015; Friedman test) was identified, which was predominantly driven by a higher rate in Nef, compared to Pol (**, p = 0.015; Holm-corrected Wilcoxon test). A trend was present when comparing the rate between Gag and Pol (p = 0.054) and Nef (p = 0.054). (TIF) [file ppat.1010965.s010.tif]

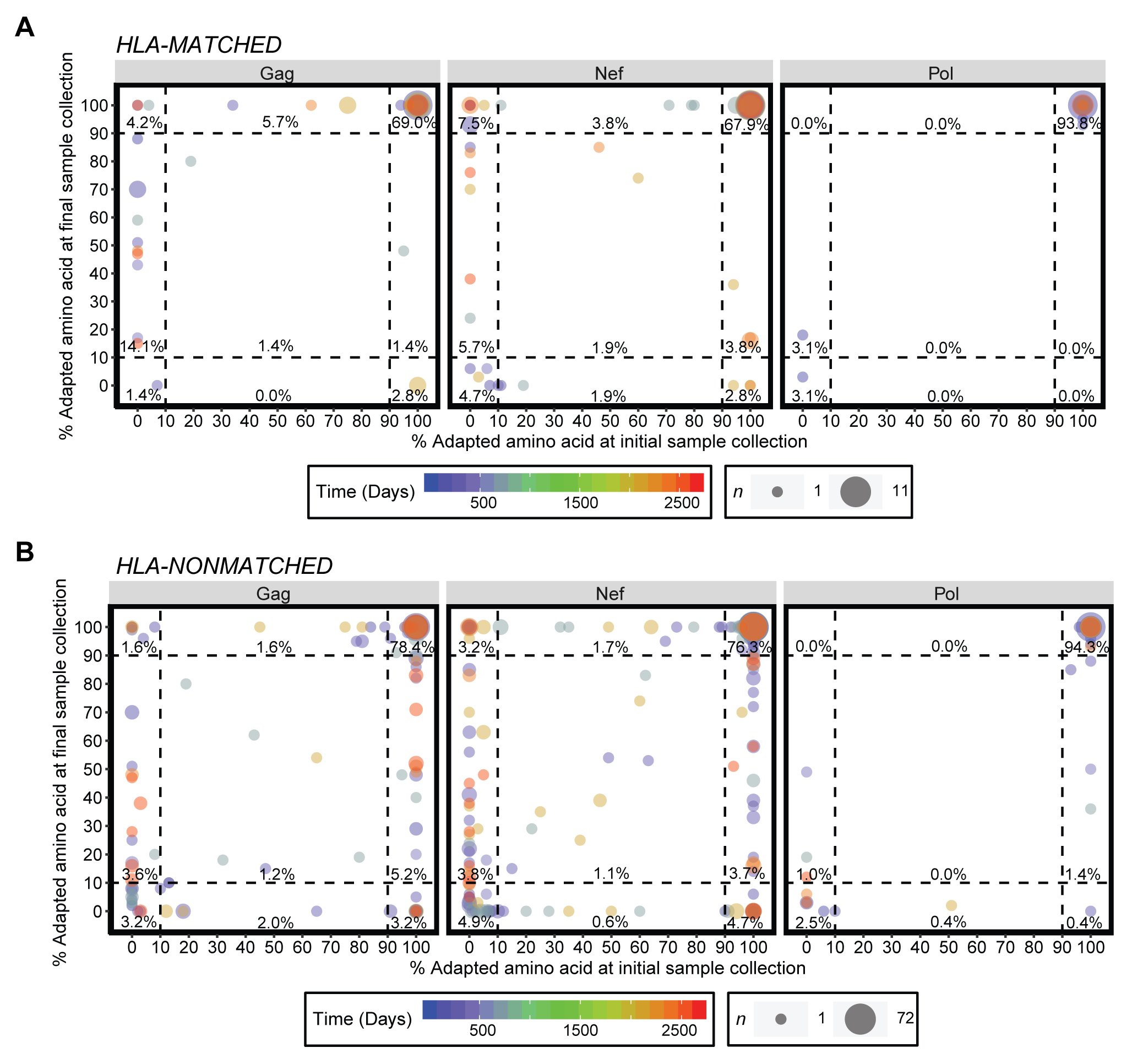

Supplement: S3 Fig — A) HLA-matched adaptation dynamics. B) HLA-nonmatched adaptation dynamics. (TIF) [file ppat.1010965.s011.tif]

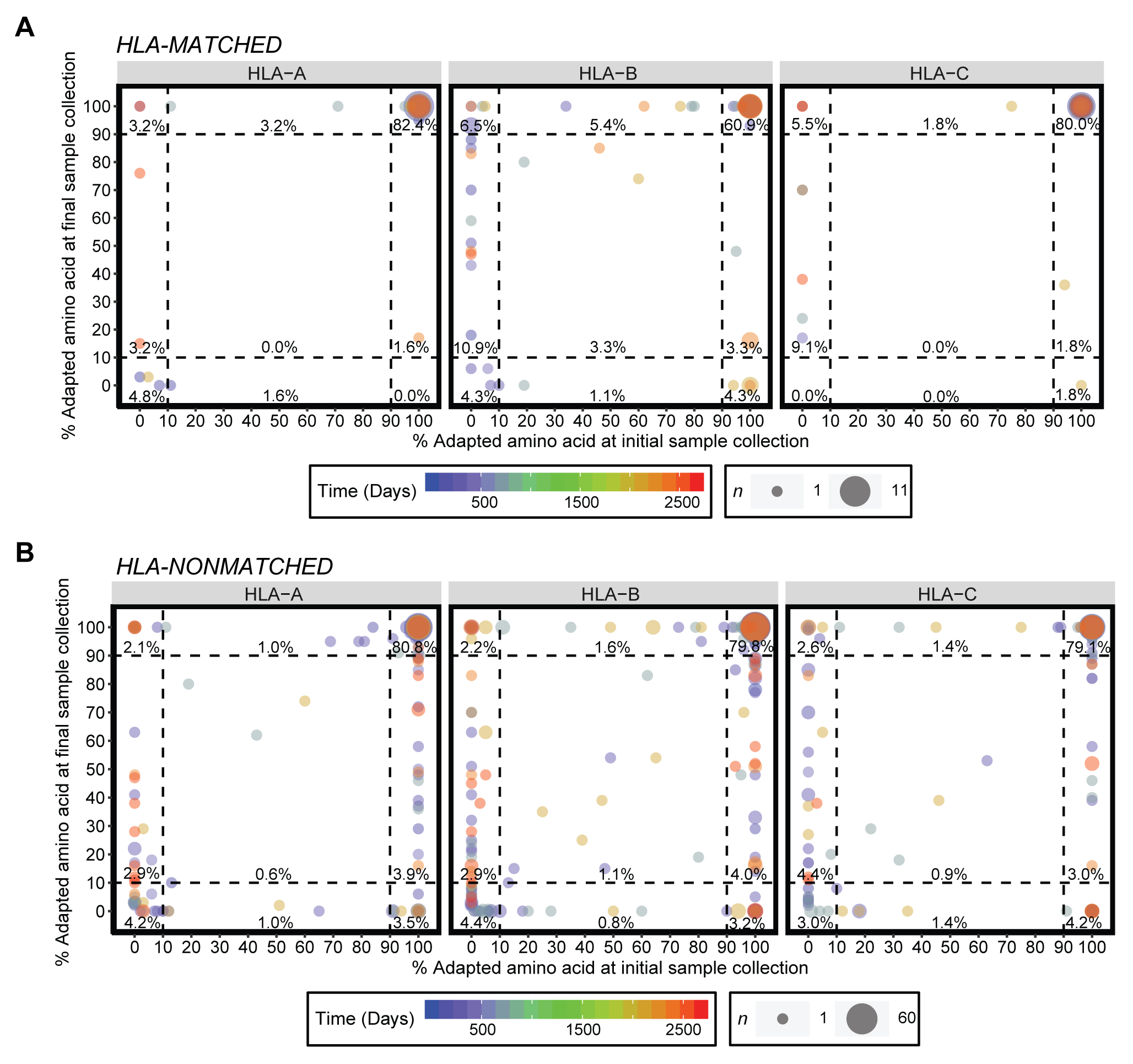

Supplement: S4 Fig — A) HLA-matched adaptation dynamics. B) HLA-nonmatched adaptation dynamics. (TIF) [file ppat.1010965.s012.tif]

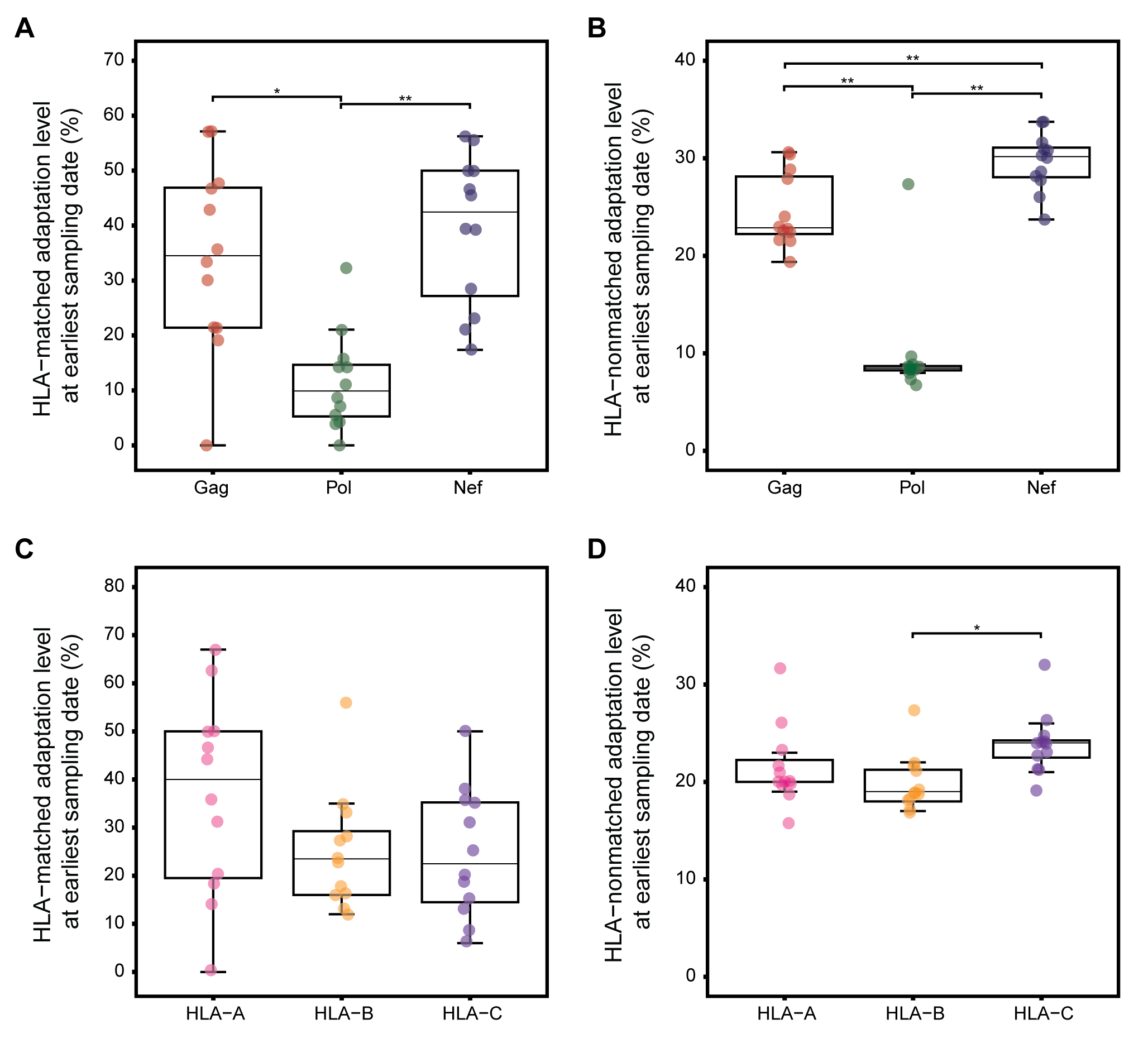

Supplement: S5 Fig — A) HLA-matched adaptation level of Gag (*, p = 0.012) and Nef (**, p = 0.004) is significantly higher than Pol and exhibits a high degree of variation between subjects. B) HLA-nonmatched adaptation is similarly significantly higher in Gag (**, p = 0.001) and Nef (**, p = 0.001), than Pol, and displays less inter-subject variability than HLA-matched adaptation. Moreover, Nef appears to have greater levels of adaptation compared to Gag (*, p = 0.005). C) No significant difference between HLA-matched adaptation level of HLA class I loci (p = 0.558). D) HLA-C has a significantly higher level of HLA-nonmatched adaptation in this cohort, compared to HLA-B (*, p = 0.017). Analyses using Friedman test and Holm-corrected Wilcoxon test. (TIF) [file ppat.1010965.s013.tif]

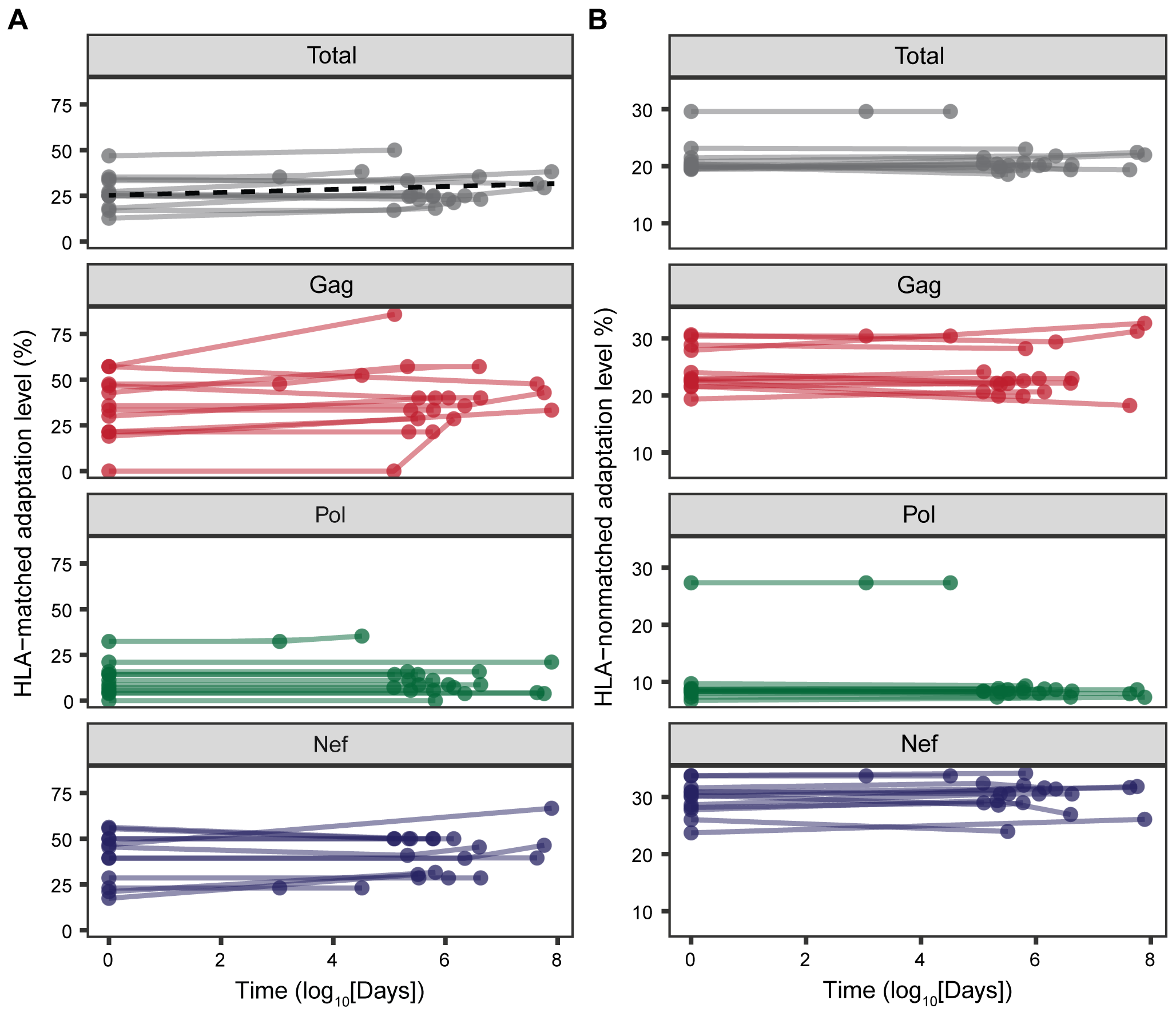

Supplement: S6 Fig — A) Changes in HLA-matched adaptation level over time by subject. Total HLA-matched adaptation level exhibited a significant positive correlation with time (p = 0.025). Whereas no significant correlations with time were identified for subject Gag (p = 0.052), Pol (p = 0.525) or Nef (p = 0.093) HLA-matched adaptation changes. B) No correlations with time were identified for total (p = 0.403), Gag (p = 0.936), Pol (p = 0.967) or Nef (p = 0.174) HLA-nonmatched adaptation level. Analyses conducted using mixed effects linear regression. (TIF) [file ppat.1010965.s014.tif]

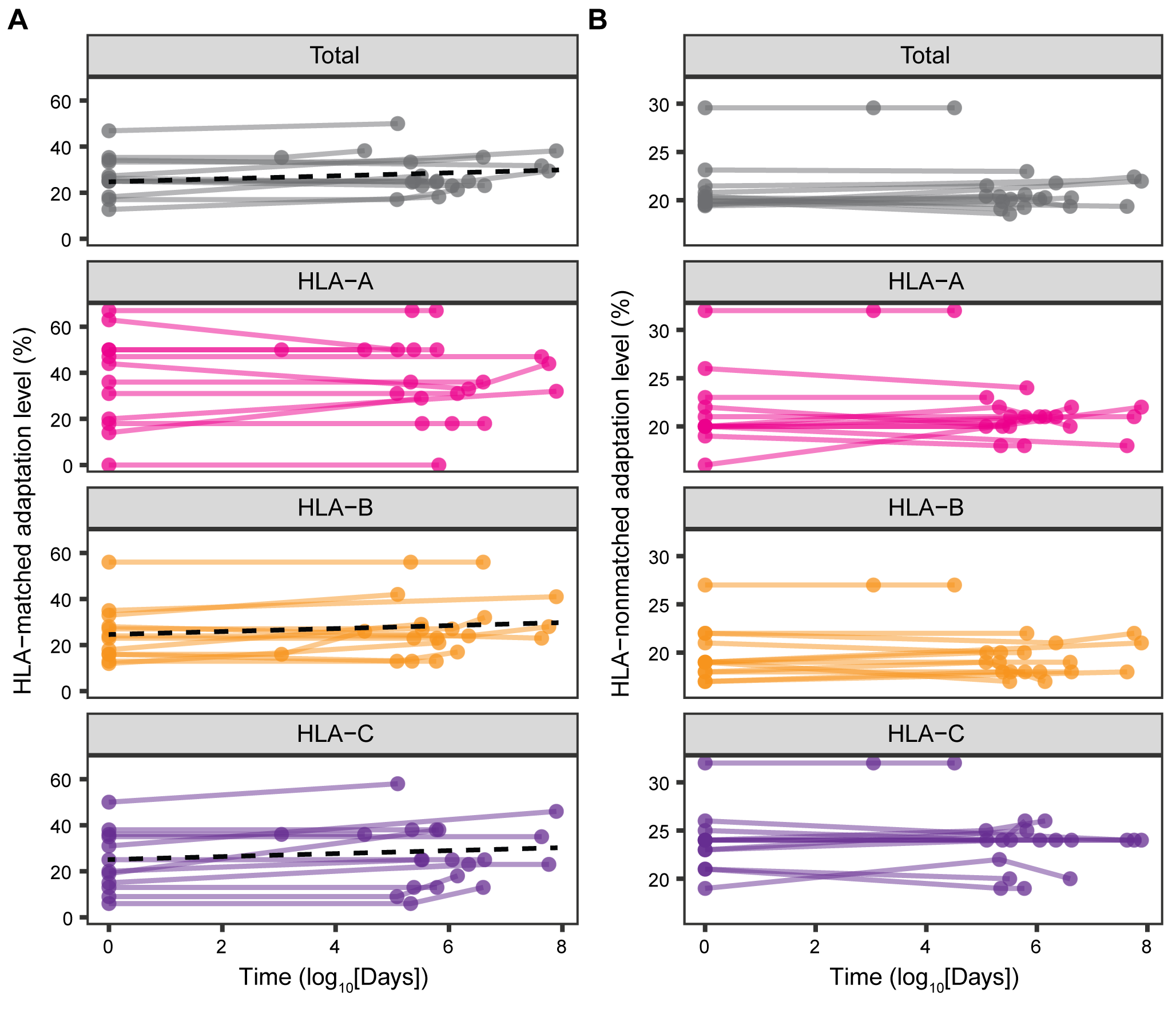

Supplement: S7 Fig — A) Changes in HLA-matched adaptation level over time by subject. Total (p = 0.025), HLA-B (p = 0.037) and HLA-C (p = 0.002) associated HLA-matched adaptation level exhibited a significant positive correlation with time. However, no significant correlation with time was identified for HLA-A (p = 0.600). B) No correlations with time were identified for total (p = 0.403), HLA-A (p = 0.531), HLA-B (p = 0.626) or HLA-C (p = 0.836) HLA-nonmatched adaptation level. Analyses conducted using mixed effects linear regression. (TIF) [file ppat.1010965.s015.tif]

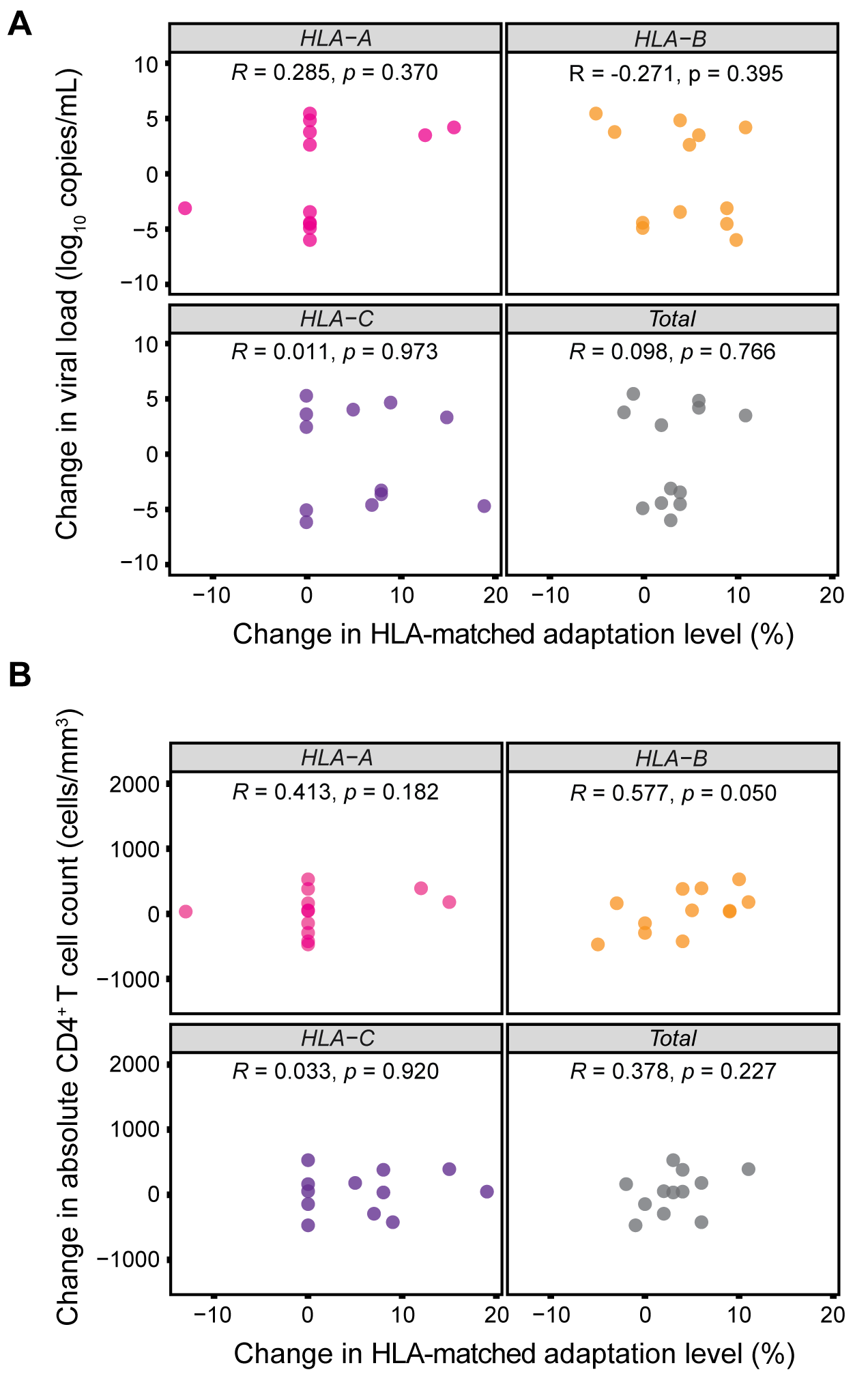

Supplement: S8 Fig — Analyses using spearman’s rank correlation rho. (TIF) [file ppat.1010965.s016.tif]

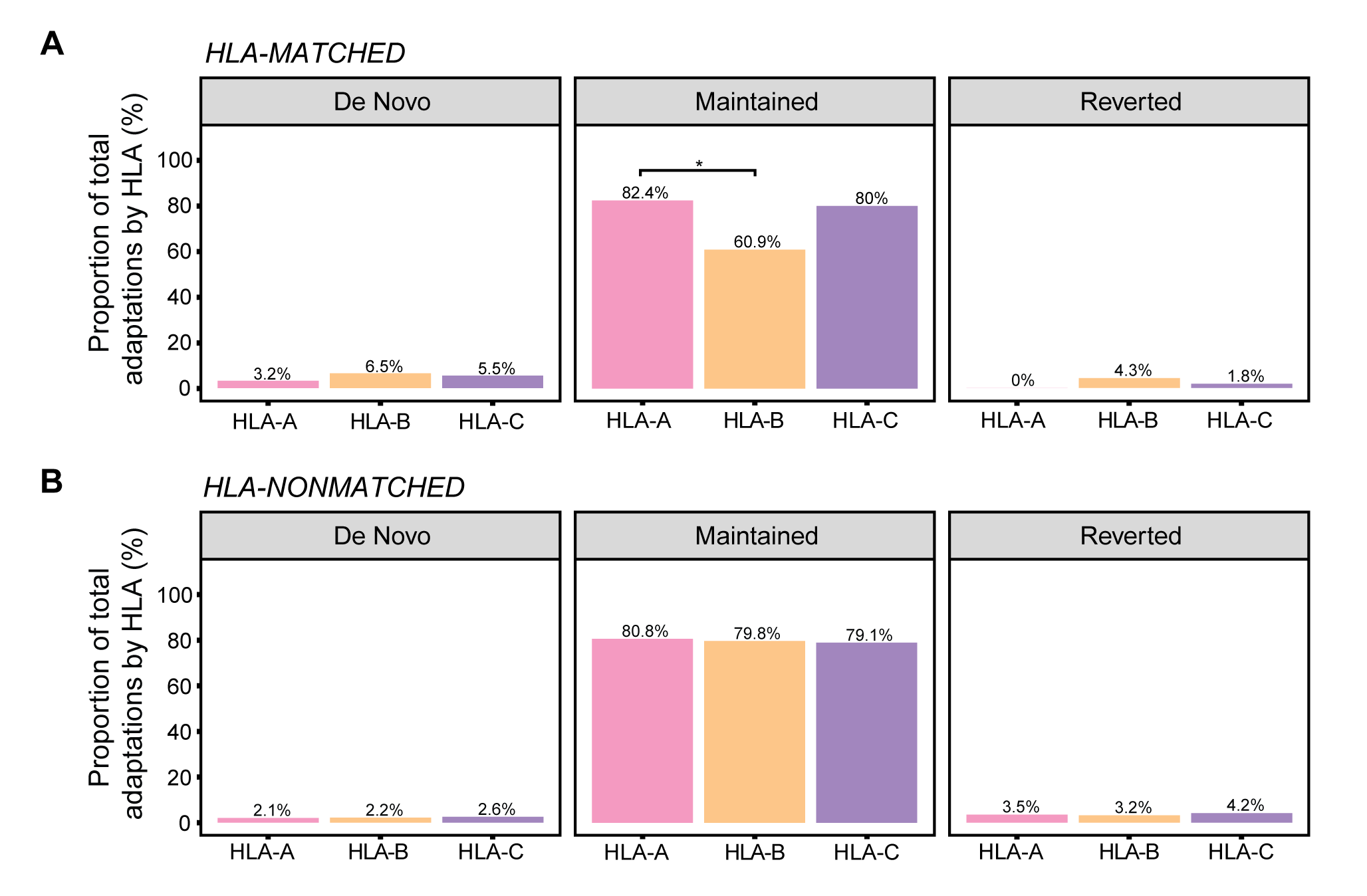

Supplement: S9 Fig — A) A significantly lower proportion of HLA-B-associated maintained adaptations were identified, compared to HLA-A-associated adaptations (*, p = 0.024), and a similar trend was identified when compared with HLA-C-associated maintained adaptations (p = 0.052). However, no difference in the proportion of de novo or reverted adaptations between HLA-associations was found. B) No difference in proportions was identified between HLA-associations in the HLA-nonmatched category. Analyses conducted using three-sample test for equality of proportions and Holm-corrected pairwise comparison of proportions. (TIF) [file ppat.1010965.s017.tif]
